# Prediction of pre- and postfusion conformations of class I fusion proteins with AlphaFold2

**DOI:** 10.1371/journal.pone.0351662

**Published:** 2026-06-16

**Authors:** Sevilay Gülesen, Victoria Most, Clara T. Schoeder, Jens Meiler

**Affiliations:** 1 Institute for Drug Discovery, Faculty of Medicine, Leipzig University, Leipzig, Germany; 2 Center for Scalable Data Analytics and Artificial Intelligence ScaDS.AI, Dresden/Leipzig, Germany; 3 Department of Chemistry, Department of Pharmacology, Center for Structural Biology, Institute of Chemical Biology, Center for Applied Artificial Intelligence in Protein Dynamics, Vanderbilt University, Nashville, Tennessee, United States of America; 4 Institute for Computer Science, Faculty of Mathematics and Informatics, University Leipzig, Leipzig, Germany; 5 Wilhelm Ostwald Institute for Physical and Theoretical Chemistry, Faculty of Chemistry and Mineralogy, University Leipzig, Leipzig, Germany; 6 School of Embedded Composite Artificial Intelligence (SECAI), Dresden/Leipzig, Germany; The Scripps Research Institute, UNITED STATES OF AMERICA

## Abstract

Viruses such as coronaviruses or filoviruses use their surface glycoproteins (GPs) to attach to the host cell, triggering the fusion of the viral membrane with the endosome membrane. Epitopes on the viral GP are major targets for antibody-mediated recognition and neutralization. During the fusion process, the GP undergoes conformational changes triggered by fluctuations in environmental pH. Structural states are typically classified into three distinct conformations: prefusion, intermediate, and postfusion. These conformations serve as essential templates for prediction of conformational epitopes and structure-based vaccine design. Despite their importance, many viral GP structures remain absent from the Protein Data Bank (PDB). Fortunately, recent breakthroughs in computational structure prediction have greatly enhanced the accuracy and accessibility of protein modeling. In this study, we utilized AlphaFold2-Multimer (AF2-M), version 2.3, to predict various GP structural conformations and observed that the overall frequency of predictions in the postfusion conformation is low. Therefore, we hypothesized that adapting the AF2-M protocol is necessary to enrich for specific conformations, thereby enabling the prediction of both pre- and postfusion conformations. AF2-M requires only the input sequence and internally generates multiple sequence alignments (MSAs) and optional templates before applying its pretrained model weights. We tested the use of template data to enrich pre- or postfusion conformations and demonstrated that our approach significantly increases the prediction frequency of class I fusion protein structures in both conformations, with the template dataset playing a crucial role in guiding modeling towards the intended state. Furthermore, we showed that the lack of correlation between pLDDT and TM-scores suggests that low pLDDT values may obscure the presence of valid alternative conformations.

## Introduction

Highly pathogenic viruses, such as Ebola virus (EBOV), Influenza, Marburg virus (MARV), and coronaviruses (SARS-CoV-2), have specialized viral surface glycoproteins (GPs) known as class I fusion proteins. These proteins mediate the fusion of the viral envelope with the host cell membrane, which is an essential step in viral entry and infection [1]. Significant conformational changes and unique structural rearrangements within these proteins are essential to drive the complex mechanism of membrane fusion. These drastic conformational changes are orchestrated by a tightly regulated interplay between host-receptor interactions and pH differences within the endosome. Understanding conformational ensembles that represent these structural states ––prefusion, intermediate, and postfusion–– is vital for the development of antiviral therapeutics and the design of effective vaccines. Conversely, the functional constraints of class I fusion proteins restrict their sequence space, thereby providing the host immune system with conserved conformational epitopes that are targeted by neutralizing antibodies to prevent viral infection or disease [2,3]. These conformational epitopes are typically discontinuous and accessible through the protein three-dimensional (3D) structure [4, 5]. For vaccine design, it has been shown that the protein must be stabilized in the prefusion state as only this conformation elicits potent neutralizing and/or protective antibody responses. In contrast, proteins in the postfusion state often induce antibody populations that lack protective efficacy [6,7]. However, molecular level structural information for the metastable prefusion state is essential for structure-based vaccine design, while information about the stable postfusion state can further support rational antigen engineering and characterization [8]. By comparing these two states, negative design approaches can be implemented to introduce mutations that either destabilize the postfusion structure or lock the protein in its prefusion conformation [2]. Furthermore, characterizing the postfusion structure helps identify non-neutralizing ‘decoy’ epitopes that divert immune response and supports the development of tools to monitor vaccine stability during storage [9,10]. Overall, structural information at the molecular-level is essential for structure-based vaccine design.

**Fig 1:**
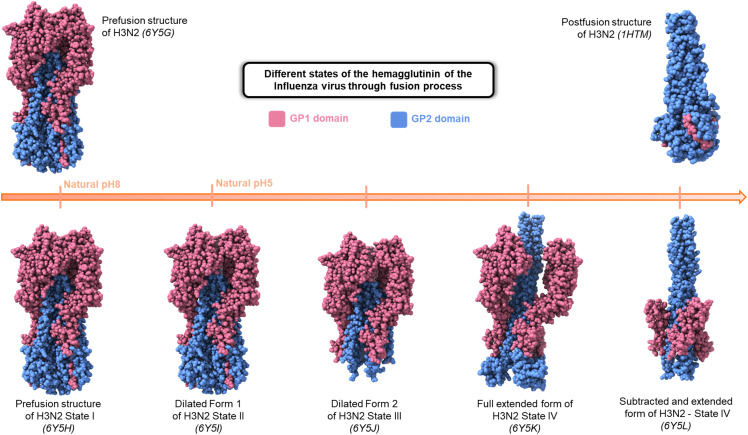
Conformational states of Influenza HA during the fusion process. Structural representations were generated by the authors using ChimeraX from publicly available PDB coordinate files. The structures correspond to PDB IDs: 6Y5G, 6Y5H, 6Y5I, 6Y5J, 6Y5K, 6Y5L, 1HTM [11–17]. The figure layout and annotations were created by the authors for illustrative purposes.

The structures of class I fusion proteins have been extensively studied with structural determination techniques such as X-ray crystallography and cryo-electron microscopy (cryo-EM) [11]. However, these techniques typically capture proteins in their most stable or metastable states, often struggle to obtain the unstable or transient intermediate conformations that occur during the fusion process [12]. Consequently, observed structures are largely restricted to the initial (prefusion) and terminal (postfusion) states, leaving the intermediate mechanistic transitions largely uncharacterized. Hemagglutinin (HA), the class I fusion protein of Influenza virus, has been studied intensively for over 50 years. Utilizing cryo-EM, researchers have successfully delineated multiple pH-dependent intermediate states, providing critical snapshots of the conformational landscape (Fig 1) [13].

For many viral GPs, the prefusion state has been reported more frequently in the Protein Data Bank (PDB) compared to the postfusion state [13]. Postfusion structures are mostly determined by X-ray crystallography [14]. For instance, while over ten prefusion state structures have been deposited for the Lassa virus (LASV) GP, only two postfusion state structures are currently available. Acquiring high-resolution structural models for both states is essential to support the requirements of structure-based vaccine design.

Recent developments in deep learning-based protein structure prediction have drastically increased modeling accuracy. Examples for such methods are AlphaFold (AF), RoseTTAFold, and OmegaFold [18–20]. These approaches have revolutionized structural biology by accurately predicting monomeric protein structures, including previously uncharacterized proteins [21]. Besides modeling monomeric protein structures, the accurate prediction of multimeric protein complexes has become an important topic of research [22]. Multimer prediction aims to estimate the interfaces, binding sites, and different conformations that occur during protein-protein interaction. Tools such as AlphaFold2-Multimer (AF2-M) are capable of modeling structures that have more than one chain with paired multiple sequence alignments (MSAs), chain-aware positional encoding, and inter-chain templates [23,24]. Thus, AF2-M provides advanced features for inferring protein complex topologies by combining co-evolutionary data, deep learning, and template-based modeling. In light of these capabilities, AF2-M has been shown to model alternative protein conformations, specifically when the input parameters are modified. In particular, adjusting the MSAs or structural templates can bias the model toward sampling distinct conformational states. Templates derived from experimentally determined PDB structures typically represent stable protein conformations and exert a strong influence on the resulting model. AF is designed to optimize for the most probable structure of a given sequence; however, it is not fundamentally aware of the underlying energy landscape. [25].

Several studies have shown to predict possible conformations of protein complexes incorporating template features of AF2. One study demonstrated that incorporating templates derived from protein language models, such as MSATransformer and ESM-1b, considerably improve prediction accuracy, particularly for proteins with shallow MSAs [26]. In that study, structure prediction performance was enhanced by integrating structural templates and MSA embeddings––a numerical vector that encodes a protein sequence. These models were pre-trained on massive protein sequence datasets to generate vectors to capture evolutionary and structural information, which were used in structure prediction to provide more context than raw amino acid sequences alone. Their approach resulted in greater prediction accuracy, particularly for challenging targets. The model outperformed established approaches such as RoseTTAFold, specifically for targets lacking high-quality templates, proving the efficacy of this integrated strategy in a variety of protein prediction benchmarks. Another study aimed to rescue low-confidence AF2 models by integrating high-confidence structural templates from the same protein family, resulting in improved outcomes even when the MSA input was disabled [27]. Davide et al. demonstrated that AF2 combined with user-defined template selection from GPCRdb and KLIFS, shallow MSAs, and optional template shuffling to different states of GPCRs and kinases by biasing models toward desired conformations [28]. These studies show that AF2 has the ability to predict different conformations of proteins, however, requires manipulation to obtain the best results. In tools such as ColabFold, these manipulations can be easily achieved [29]. ColabFold builds on AF2’s code but accelerates predictions by using MMseqs2 for fast MSA generation, making it much quicker than the original [18,29]. It supports custom MSAs, batch processing in Google Colab, multimer modeling, and adjustable accuracy settings, while also allows users to supply specific templates to enhance prediction quality [30].

In this study, we developed a template-driven modelling approach to capture both the pre- and postfusion conformations of viral GPs. Our strategy is based on the hypothesis that the conformational states of class I fusion proteins can be predicted within ColabFold framework by carefully selecting the templates provided to the method [29]. Furthermore, this study aimed to model real-world GPs lacking experimentally determined structures in the PDB, representing a typical use case for vaccine design. To evaluate these approaches, we prepared two different benchmark sets of class I fusion proteins. The first benchmark set was used to validate the prediction accuracy, while the second benchmark set covered sequences of real-world GPs with no available structural data. Our results demonstrated that the selection of distinct templates has a direct and measurable impact on the resulting conformational states.

## Results

### Two different benchmark sets were prepared for class I fusion protein structure prediction

First, a benchmark set detailing pre- and postfusion structures was assembled from the PDB. Most of the structures were prefusion structures in the PDB when compared to other conformational states as of the time of this study [31]. The major challenge here was that the AF2 training set contained many of these structures, while only few structures were not deposited by the time of the AF2 training release (2021-09-30) [32]. Therefore, two different benchmark sets were prepared, which 1) contained only canonical (classical) class I fusion proteins, whose structure had been determined and 2) a set of unknowns which were used as a performance test case. The first benchmark set named canonical benchmark set, consisted of the GPs of Influenza virus (hemagglutinin, HA), respiratory syncytial virus (RSV), Ebola virus (EBOV), Marburg virus (MARV) and Lassa virus (LASV) (Table 1).

**Table 1:** PDB codes of selected structures of the canonical benchmark set.

Virus	Prefusion PDB	Postfusion PDB
EBOV	3CSY [33,34]	2EBO [35,36]
HA	1HGG [37,38]	1HTM [39,40]
LASV_1	7PUY [41,42]	5OMI [43,44]
LASV_2	5VK2 [45,46]	6JGY [47,48]
MARV	6BP2 [49,50]	4G2K [51,52]
RSV	4MMS [8,53]	3RKI [54,55]

As we have a special interest in modeling arenavirus GPCs in our laboratory, two sets of structures for the LASV GPC were selected from the Mouse/Sierra Leone/Josiah/1976 strain. Both sequences of these selected LASV GPCs originated from the same strain but their sequences were not identical and included different mutations and gaps in the deposited structures. The pre- and postfusion structures were matched from the same strain for every virus and cropped to the sequence coverage and tags (S1 Table). This canonical benchmark set was used to determine the performance of the proposed protocol. As a second benchmark case, we wanted to evaluate the performance if both structures were not available yet, representing a typical use case in vaccine design. In this second benchmark set (also called real-world benchmark set), arenaviruses GPCs were selected including Junín virus (JUNV), Machupo virus (MACV), Lujo virus (LUJV) and Chapare virus (CHAV) (S2 Table). As of the experiment day, most of these viruses’ crystal structures have not been deposited for either prefusion or postfusion structures in the PDB [14]. To our advantage, the prefusion structure of JUNV, MACV and LUJV GPC was reported recently and could be used as a reference structure for comparison of our predictions. Since an experimentally determined postfusion structure of JUNV GPC was not available, and in order to facilitate a comprehensive comparative analysis, the real-world benchmark set was initially evaluated against the pre- and postfusion conformations of the LASV GPC. Additionally, the JUNV, MACV and LUJV prefusion structures were examined separately to provide a more detailed comparative analysis by using their experimentally determined prefusion structures. For both benchmark sets, the same template structures were utilized (S3 Table), and the reference PDB files of every GPs in the canonical benchmark set were excluded from their own templates and only included as templates of other viruses. All strategies—default, all-templates, prefusion-templates and postfusion-templates— were tested using the chosen sequences for the canonical and real-world benchmark sets.

All four modeling strategies—default, all-templates, prefusion-templates, and postfusion-templates—were initially applied using the full-length sequences for both the canonical and the real-world benchmark sets; this approach is hereafter referred to as the full-length sequence approach (S1 Fig). However, previous literature has established that the GP1 subunit is typically cleaved and not retained in postfusion conformation, which is primarily driven by the refolding of the GP2 subunit. This mechanism is consistent with the general function of class I viral fusion proteins (with the exception of RSV F protein). Accordingly, we implemented an additional strategy, designating the cropped-sequence approach, specifically for modeling the postfusion conformation (S2 Fig.). This involved utilizing only the GP2-derived sequences (S4 and S5 Table). All four modeling strategies (default, all-templates, prefusion-templates, and postfusion-templates) were then reapplied using these cropped (only GP2) sequences for the benchmark sets.

### Templates informed ColabFold to predict pre- and postfusion states reliably

**Fig 2:**
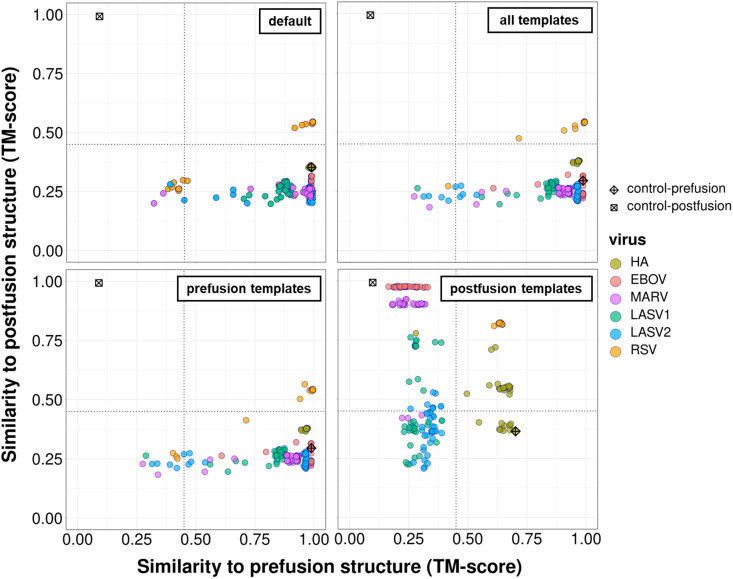
TM-score analyses of the canonical benchmark set obtained using the full-length sequence approach. Results of four strategies as default, all-templates, prefusion-templates and postfusion-templates, using the full-length sequence approach are shown. TM-score >=0.45 signifies similar overall structural topology.

Four different strategies were chosen for prediction of pre- and postfusion conformations of our canonical benchmark set (Table 1). As a first strategy, ColabFold was executed with default inputs for the respective sequences. All other strategies included specified template options. Three template collections were generated which contained all-templates (all pre- and postfusion structures), only prefusion-templates and only postfusion-templates, while omitting the respective test case structures. In the template strategy, ColabFold was limited to use only provided templates which can be compared to an AF-informed homology modeling approach. Since the sequence of prefusion structures differs in length from the sequence of the postfusion structure (except RSV), structural similarity was determined with a TM-score analysis rather than RMSD (Root Mean Square Deviation). TM-scores of all predicted structures were calculated with their known pre- and postfusion structures as a reference structure mentioned in Table 1. As another check for the predicted structures, the control runs were generated with the exact sequences of the reference structures. Then, results were compared with their own structures through calculation of the TM-score, and the best TM-score was selected for each case among all viruses TM-scores above 0.45 were considered to represent acceptable structural matches, whereas values approaching the control threshold were interpreted as indicating high structural similarity. Overall, the prediction of the prefusion state was accurate for the majority of the canonical benchmark set. Three out of four strategies yielded highly comparable results (Fig 2). In the default, but also with the all-templates and prefusion-templates only, ColabFold was not able to predict any postfusion structures of any viruses. For these three cases, only prefusion structures were predicted, and resulted in TM-scores distributed around or over 0.45. This suggests that the predicted exhibit substantial structural similarity to the reference structure and can therefore be regarded as matching predictions. In the postfusion-templates strategy, ColabFold predicted the postfusion conformations successfully for some GPs, but not all. Especially, most predictions for EBOV GP and MARV GP were populated as postfusion structures. It has to be noted that the PDB contains multiple postfusion structures for EBOV and MARV GPCs, therefore, it might be that ColabFold maintains residual memory for these. Accordingly, this indicates that ColabFold has a capability to model the postfusion conformation, but with overall low accuracy. For other GPs, results were distributed in a wide range. TM-scores of LASV1 and LASV2 GPs were mostly scattered below 0.45, which means models were not identified as prefusion or postfusion states. ColabFold modeled acceptable postfusion structures for HA, although all models were closer to the prefusion reference than the expected postfusion conformation –but visual inspection showed that the structures were similar with intermediate states of HA. For RSV F, models were obtained which clustered with the prefusion structure for the default, all-templates and prefusion-templates strategies, and more strongly with the postfusion for the postfusion-templates strategy. It is important to note that the postfusion structure of RSV F is similar to its prefusion structure, because it does not lose its membrane distal subunit. Therefore, the TM-score separation between the prefusion and postfusion predictions for RSV F was smaller.

**Fig 3:**
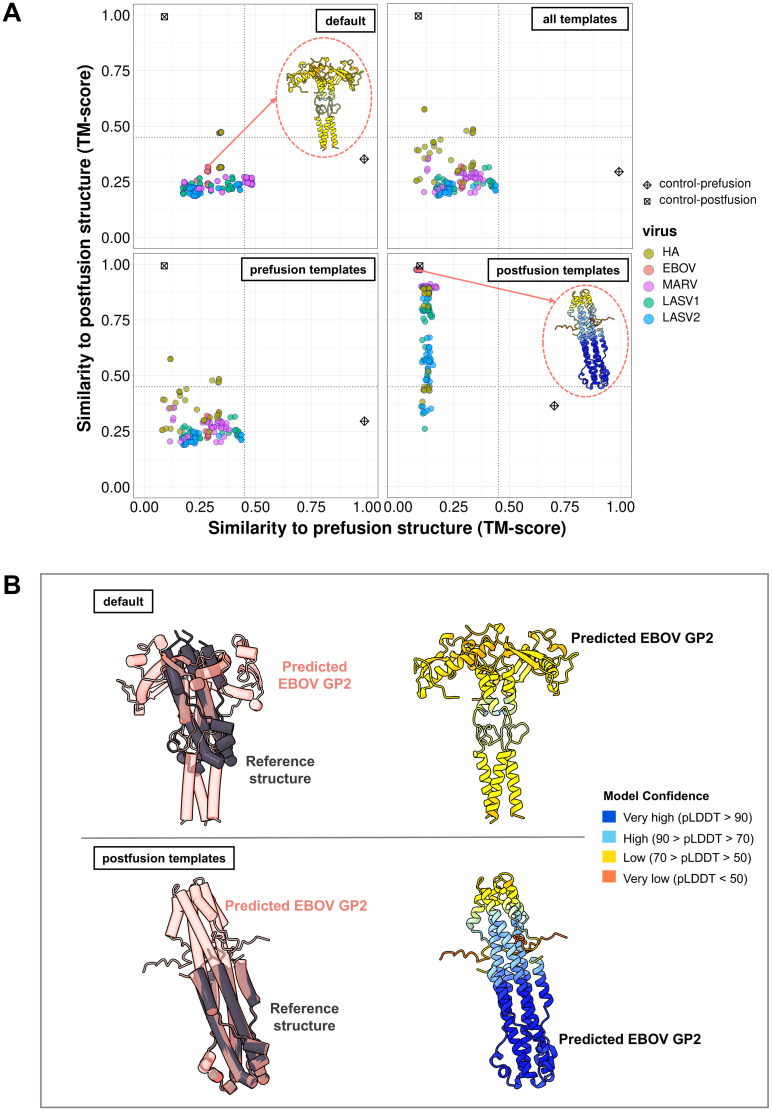
TM-score analyses of the canonical benchmark set obtained using the cropped sequence approach. **(A)** TM-score analyses of the canonical benchmark set according to four strategies as default, all-templates, prefusion-templates and postfusion-templates, using the cropped sequence approach. The models that have the best TM-scores for the default and postfusion-templates strategy were shown based on EBOV as an example. TM-score >=0.45 signifies similar overall structural topology. **(B)** The selected EBOV GP models were aligned with the postfusion reference structure (PDB ID: 2EBO) shown in grey.On the left, the predicted models are shown indistinct transparent colors to visualize the structural alignment. On the right side,the same models are colored according to per-residue pLDDT values; orange, 0–50; yellow, 50–70; cyan, 70–90; and blue, 90–100 [35]. The structural visualizations were generated by the authors using ChimeraX from AF2-M models produced in this study and publicly available PDB coordinate files used as reference structures.

Many structural conditions of the fusion process are still unknown. For most of the postfusion GPs structures in this set, the GP1 subunit is not visible after the fusion process, and the postfusion structure requires only the presence of the GP2 subunit (except RSV F). Biologically, it is unclear what happens to GP1 during the fusion process [56]. However, the structural rearrangement should ensure that there are very few co-evolutionary contacts between GP1 and GP2 as there is not much structural dependence on the subunits on each other [57]. Therefore, the postfusion structures were predicted with cropped sequences only representing GP2. The same four strategies were applied with the previously described templates to predict especially a postfusion state using only the GP2 sequences. Results showed that for the default, all-templates and prefusion-templates strategy, ColabFold predicted neither the postfusion conformation but also not prefusion conformation either (Fig 3A). In the postfusion-templates strategy, ColabFold was able to predict postfusion structures for all GPs. Most predictions yielded postfusion structures with high TM-scores in 190 trajectories. RSV F was not included in this test because GP1 and GP2 remain linked in its postfusion state.

To investigate whether the best models by the TM-score, presenting the most accurate predictions would also be identified by the ColabFold as the models with the highest confidence, the results were ranked according to the ColabFold pLDDT (the predicted Local Distance Difference Test) score. The default strategy did not align with the postfusion reference structure and the confidence scores (that was calculated residue based) were lower than 70 (Fig 3B and S3), clearly showing that both scoring methods consider this model to be less reliable.

### Predictive power for the real-world benchmark set is better than the canonical benchmark set

As the ColabFold informed by either prefusion or postfusion templates was able to produce structures in pre- and postfusion conformation for most of our benchmark set, we moved to predict our real-world test set. Sequences were both prepared as full-length sequence and as cropped GP2 only sequence.

**Fig 4:**
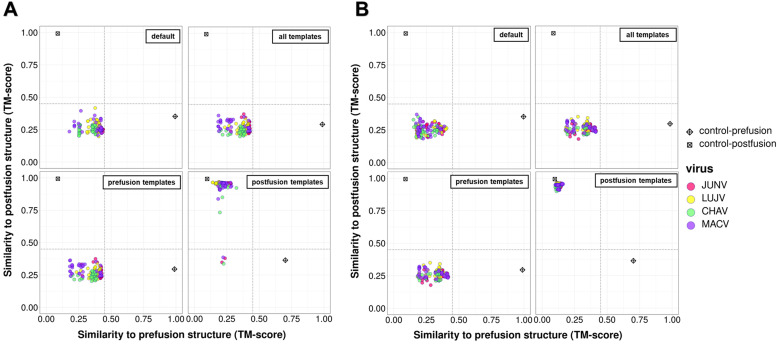
TM-score analyses of the real-world benchmark set. Results of four strategies as default, all-templates, prefusion-templates and postfusion-templates are shown. Pre- and postfusion states of the LASV GPC were used as reference structures. TM-score >=0.45 signifies similar overall structural topology. **(A)** TM-score results for the full-length sequence approach are represented. **(B)** The cropped sequence approach predictions are shown.

Again, all four strategies were applied to predict the unknown structures. Predicted structures were compared with the LASV pre- and postfusion structures, as the crystal structures of prefusion states for the respective GPs for the arenaviruses in our real-world benchmark set had not been established at the time of the study. Additionally, postfusion structures have yet to be determined through experimental means. Therefore, the pre- and postfusion structures of the LASV GPC used as the reference standard to facilitate a comprehensive comparative analysis. For our four strategies both with the full-length and GP2 only sequences, we obtained low TM-scores of the real-world benchmark set for the prefusion-state prediction (based on the LASV GPC) (Fig 4). However, the postfusion-state predictions obtained with the postfusion-templates strategy yielded high TM-scores, closely matching the control-postfusion point for every arenavirus in the test set. (Fig 4A). When comparing results of the full-length and cropped sequences for the postfusion-templates strategy, the cropped GP2 only sequences had higher scores than the full-length (Fig 4B). In fact, this produced a substantially higher yield of postfusion structures compared to our initial benchmark set.

**Fig 5:**
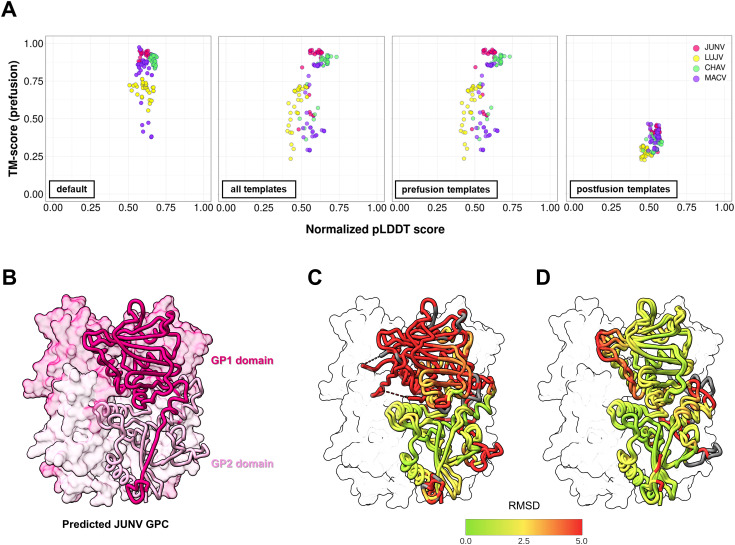
Analyses of the real-world benchmark set. **(A)** TM-score and pLDDT score analyses of the real-world benchmark set according to the four strategies as default, all-templates, prefusion-templates and postfusion-templates with the full-length sequence approach. TM-score analyses for the prefusion-states were conducted using their respective experimentally determined prefusion structures, except for the CHAV analysis, which utilized the JUNV structure due to the absence of an experimentally determined prefusion state for the CHAV. TM-score >=0.45 signifies similar overall structural topology. pLDDT score is considered as; very low, 0–50; low, 50–70; high, 70–90; and very high, 90–100. **(B)** Structural representation of the top-ranked model predicted by the prefusion-templates strategy. The GP1 domain is colored magenta, and the GP2 domain is colored light pink. **(C)** RMSD analysis of the top-ranked JUNV model generated using the prefusion-templates strategy by comparing with the LASV GP reference structure (PDB: 7PUY). **(D)** RMSD analysis of the top-ranked JUNV model generated using the prefusion-templates strategy by comparing with the JUNV GP reference structure (PDB: 9GHJ). Structures are colored by per-residue RMSD scores, classified as low (0–2.5 Å) or high (>2.5 Å), and grey residues represents missing values [41,58]. The structural visualizations were generated by the authors using ChimeraX from AF2-M models produced in this study and publicly available PDB coordinate files used as reference structures.

To investigate the prefusion state further, we assessed prefusion TM-scores against their respective experimental structures (S6 Table). For the CHAV, the JUNV GPC prefusion structure was taken as reference due to the lack of an experimental structure and the close phylogenetic relationship. Overall, the first three strategies for the full-length sequence approach were able to predict prefusion state (Fig 5A). For the postfusion-templates strategy, the results clearly showed that prefusion state had not predicted successfully, which was expected due to structural differences. This was also the reason for the fact that all four strategies in the cropped sequence approach did not predict the prefusion conformation (S4 Fig).

The top-ranked model generated by the prefusion-templates strategy is presented in Fig 5B, distinguishing the GP1 and GP2 domains. This model was evaluated via RMSD analysis against the LASV and JUNV GP reference structures. Superposition of the best model with the LASV GP revealed limited structural similarity (Fig 5C). Although the GP2 domains appeared similar with low per-residue RMSD scores, the GP1 domains were distinct, as evidenced by high RMSD values. This observation aligns with the previously discussed TM-score results. In contrast, when superimposed onto the experimental JUNV GP prefusion structure, the model exhibited per-residue RMSD values predominantly below 2.5 Å, indicating high accuracy (Fig 5D). The correct folding of both the GP1 and GP2 domains in this comparison is consistent with the high TM-scores.

### pLDDT may not be indicative for modeling performance for class I fusion proteins

For both the full-length and cropped sequence approaches, pLDDT scores were analyzed based on the default, all-templates, prefusion-templates and postfusion-templates strategies. According to the normalized pLDDT (pLDDT scores divided by 100 to scale values between 0 and 1) of the canonical benchmark set, the prefusion comparison results of the full-length sequence approach were mostly distributed over 0.7 (which is equal to 70 for a normal pLDDT score) for three out of four strategies, which can be considered high confidence for the models (S5 Fig). When looking at the TM-score analyses, these high pLDDT scores were expected. ColabFold models of the full-length strategy for these three strategies were mostly clustered as a prefusion-state because the TM-scores were high for the prefusion conformation and low for the postfusion conformation. It is important to acknowledge that AF2-M was trained with the dataset that contains mostly prefusion structures. Thus, that might be why the confidence score was high for these cases.

**Fig 6:**
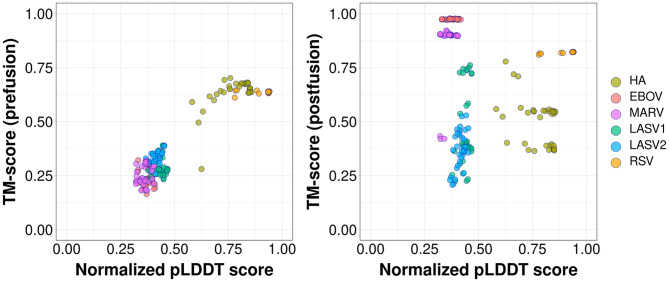
Normalized pLDDT score and TM-score analyses of the canonical benchmark set. The results of the postfusion-templates strategy with the full-length sequence approach is shown. TM-score >=0.45 signifies similar overall structural topology. pLDDT score is considered as; very low, 0–50; low, 50–70; high, 70–90; and very high, 90–100.

On the other hand, the postfusion-templates strategy was not successful in predicting the prefusion conformation, which is consistent with the correlation observed between the prefusion TM-score and pLDDT (Fig 6). This outcome may again be attributable to the AF2-M training dataset. However, comparison with the postfusion conformation revealed a lack of consistent correlation between the two metrics. The predicted models with low pLDDT values achieved high TM-scores according to the postfusion-state, suggesting that low predicted confidence may obscure alternative conformational states.

For the cropped sequence approach, the normalized pLDDT results were mainly below 0.7 (S6 Fig). When considering their TM-scores, low pLDDT scores were consistent. However, for the postfusion-templates strategy, results of the confidence analyses did not match with their TM-scores calculated based on the postfusion-state. Only the pLDDT score of the EBOV GP and some of the HA models for the postfusion-templates strategy overlaped with similarity analyses.

The TM-scores analyses of the real-world benchmark set for the full-length and cropped sequence approaches were first performed based on the LASV GPC as mentioned previously. The confidence scores of these analyses for three out of four strategies—as well as the prefusion prediction of the postfusion-templates strategy—remained primarily below 0.7 for both the full-length and cropped sequence. These low pLDDT scores correlated with low TM-scores (S7 and S8 Fig). Conversely, while the postfusion strategy of the full-length approach accurately predicted the postfusion conformation (indicated by high TM-scores), it still yielded pLDDT scores below 0.7, classifying the models as low confidence. Notably, this pattern did not align with the TM-score analyses, an outcome that was unanticipated. Similarly, in the postfusion-state predictions generated using the postfusion-templates strategy with the cropped sequence approach, normalized pLDDT values were distributed predominantly below 0.7, despite the corresponding models achieving high TM-scores.

In the detailed assessment of prefusion conformations in the real-world benchmark set, using their corresponding experimental structures, the confidence scores for all cropped sequence strategies—as well as for the postfusion-templates strategy in the full-length approach—were broadly below 0.7, consistent with their low TM-scores (Fig 5 and S4 Fig). In contrast, the default, all-templates, and prefusion-templates strategies of the full-length approach predominantly achieved high TM-scores, indicating successful prediction of the prefusion conformation, despite confidence scores remaining mainly below 0.7. Thus, an unexpected discordance between TM-score and pLDDT was observed.

## Discussion

In this work, we developed a protocol to predict pre- and postfusion conformations of class I fusion proteins using ColabFold, for which we had occasionally observed postfusion models from time to time before. However, this outcome was not frequent and not controllable by the user. We worked on manipulating the template option of AF2-M, version 2.3, in this study, as it has been shown that MSA modification alone might not be sufficient for this protein class [59,60]. They often have the limited sequence homologs for certain conformations, and the PDB contains an unbalanced representation of their pre- and postfusion states. Large unstructured regions, extensive glycosylation, and high structural variability can reduce MSA quality and complicate alignment-based predictions. For some viruses, limited sequence diversity results in weaker co-evolutionary signals, which can hinder accurate multimer or conformational modeling [61]. Although it can accurately predict the folding of monomeric subunits, predicting inter-chain interactions is frequently hindered by a lack of co-evolutionary data and limited experimental information on protein complexes. These results in multimeric predictions being less reliable and more challenging to validate compared to single-chain predictions [62].

It was reported that templates improve the accuracy of predicted structures and enable them to predict other conformations [27,28,63]. Here, we showed that incorporating templates substantially improves GP conformational modeling, with performance increases exceeding half of the template-free baseline, particularly for prediction of the postfusion conformation. Our results indicate that providing specific templates can significantly alter and enhance the predicted conformations. Also, the AF2 database contains GP structures especially, which biases the prediction for specific viral GPs.

In the case where the real-world benchmark set was used, results were compared with the LASV GPC, since there were not any published postfusion structure in the PDB for any GPs in the real-world benchmark set. Therefore, the LASV GPC was chosen as a single reference to ensure consistent comparison between the prefusion and postfusion conformations and has the most structural similarities out of the examples in the canonical benchmark set [64]. Based on the results, our approach reached high accuracy to predict postfusion-states for the real-world benchmark set, even though they compared with the LASV GPC. The reason might be that GP2 is the most conserved domain across arenaviruses which has been shown in previous studies [48,65–70]. The JUNV and LASV GP2 subunits fold into an almost identical post-fusion 6-helix bundle, as do other class I viral fusion proteins, based on structural investigations [48]. Therefore, these conserved regions might increase the accuracy to predict postfusion conformation rather than the prefusion. On the other hand, the prediction of the prefusion-state could not be successfully observed based on TM-score analyses of the LASV GPC. Therefore, the prefusion-state analyses were conducted with the prefusion structures of the LUJV, MACV and JUNV GPCs were experimentally determined after the study of benchmarking which also means that AF2 database did not contain them. In comparison of these TM-score results with the original prefusion structures, the prefusion-state predictions generated using the full-length approach across the four strategies yielded higher TM-scores relative to their LASV GPC reference results.

The pLDDT versus the TM-score analysis indicates that the predicted confidence scores do not always align with structural accuracy metrics like TM-score. In some cases, high pLDDT values corresponded to low TM-scores, while low pLDDT values were associated with high TM-scores. Even though possible postfusion conformations were sampled, AF2-M has low pLDDT scores based on the predictions. It is important to note that AF2-M was trained on a dataset predominantly composed of prefusion structures, which may explain why the confidence scores were higher for predictions in the postfusion conformation. On the other hand, although the prefusion conformations of the real-world benchmark set were correctly predicted—as demonstrated by the high TM-scores relative to their corresponding prefusion structures—the associated confidence scores remained low. This clearly suggests that the pLDDT can sometimes obscure accurate interpretation of the results, highlighting the importance of using multiple evaluation metrics to assess model quality.

The lack of structural similarity between the prefusion-state of the real-world benchmark set and the LASV GPC prefusion state puzzled us and we further investigated this. The GP1 and GP2 subunits have been investigated across arenaviruses, and it was observed that the GP2 of arenaviruses was more conserved than the GP1 [48, 65–70]. Supporting this, the main difference between the predicted models in the real-world benchmark set and the LASV GPC reference structure was observed on the receptor binding subunit (GP1), rather than the core fusion subunit (GP2). Superimposition showed that localized displacements were evident in the GP1 surface loops and connecting segments. These precise shifts in conformations might be directly responsible for the lower TM-scores. Additionally, the RMSD comparisons of the JUNV with the LASV and with its own experimentally determined prefusion structure highlight the importance of using virus-specific reference structures. In the JUNV–LASV comparison, the RMSD values for the GP1 were above 2.5 Å, indicating that such models are not sufficient for direct atomic-level structure-based vaccine design. Deviations of this scale may influence residue positioning, side-chain orientation, and epitope geometry, which are critical for rational antigen engineering [2]. In contrast, the JUNV–JUNV comparison showed lower RMSD values, emphasizing the importance of experimentally determined structures of the respective viral glycoproteins for final vaccine-design applications.

Our study mainly focused on class I viral fusion proteins, and the feasibility of our template-guided approach for class II and III fusion proteins remains untested. Class I, II, and III fusion proteins differ from one another in terms of fold, oligomeric organization, and fusion mechanism [71], and it is therefore not straightforward to assume that an approach optimized for class I proteins would translate directly to other classes. Furthermore, some viral glycoproteins do not conform to any of the three canonical classes, representing even more challenging cases for template-guided prediction. Ultimately, the success of our approach will depend on the availability of suitable structural templates and the degree of structural conservation between the template and the target protein. Similarly, intermediate conformations were not extensively examined in this study, as experimentally determined intermediate-state structures are limited and not consistently available for the viral glycoproteins analyzed here. Since these states are transient, their detection and validation remain challenging, however, they can provide important insights into fusion mechanisms and antibody targeting. As more experimentally validated intermediate structures become available, our strategy could be expanded to investigate such conformational states in future studies.

Moving beyond the approach of this study, other methods are now avaliable that might enhance performance, e.g., AlphaFold3, Boltz-1 or Chai-1 [72,73]. However, we used AF2-M over Boltz-1 because AF2-M can effectively capture inter-chain interfaces and overall topology. On the other hand, the Chai-1 method emphasizes energy-based refinement and is not inherently well-suited for predicting large-scale conformational changes or protein–protein interactions from scratch [72]. This limitation might make it less effective for capturing both pre- and postfusion conformations without additional guidance, as Chai-1 cannot directly use full template coordinates like AF and instead relies on constraint features—such as contact maps, distance restraints, or docking geometry—derived from known structures [72]. However, when combined with state-specific restraints or engineered sequence variants, it might be valuable for obtaining consensus predictions and comparing the sensitivity of predicted states to input perturbations. In contrast to AF3, AF2-M is specially designed for predicting multimeric complexes, demonstrating strong performance in identifying stable multimeric assemblies, such as viral GP trimers in their prefusion-state. In contrast, AF3 has been optimized to handle various biological molecules like DNA, RNA, and ligands, and have enhanced performance on prediction of antibody-antigen interfaces [74]. However, its effectiveness in modeling large conformational shifts or viral fusion transitions remains under evaluation, and we do not expect a significant enhancement beyond the performance of AF2-M. Similar to our approach here, we expect that additional sampling and manipulation would be necessary.

## Conclusion

In this study, we demonstrated that our method accurately predicts both the pre- and postfusion structures of the class I fusion glycoproteins with high accuracy. The template dataset plays a crucial role in directing modeling towards the intended conformation. We further observed that predicting glycoprotein structures remains challenging, particularly when experimental data are unavailable and structural knowledge is limited; in such cases, the lack of close homologs often results in low-confidence or poorly folded models. Besides that, the representation of pre- and postfusion structures within the template benchmark set was disproportionate due to the absence of suitable experimentally determined postfusion structures. Our results indicate that incorporating template information allows to guide predictions toward alternative conformations; furthermore, increasing the number of available templates tends to improve prediction outcomes. Moreover, the observed absence of correlation between the pLDDT and TM-scores suggests that low pLDDT values may mask possible alternative conformations, when selecting the model according to its pLDDT score. Subsequently, AF2-M or other methods will have to be updated including more of the most recent viral GPs to enhance performance.

## Materials and methods

### Determination of benchmark datasets

The canonical benchmark set was created using at least one pair of prefusion and postfusion structures of GPs from EBOV, HA, LASV, MARV and RSV. 6 prefusion and 6 postfusion sequences were obtained (S1 Table). GPC sequences of JUNV, MACV, CHAV and LUJV were obtained from the NCBI database for the real-world benchmark set (S2 Table) [75]. 23 prefusion and 9 postfusion structures were selected from the GPC of EBOV, HA, LASV, MARV and RSV in the PDB for the template benchmark set, ensuring that each virus was represented by at least one postfusion structure (S3 Table). For the sequences, please check SI or GitHub repository https://github.com/schoederlab/AF2_Conformations

### Protocol details

Structure predictions were performed using ColabFold version 1.5.2 (32359508e7890916a51b1680594418a78162d01c) [29]. In the default strategy, predictions utilized the standard ColabFold_batch features without supplying any external template structures. For the template-based strategies, the template benchmark set was clustered into all-templates, prefusion-templates, and postfusion-templates. To prevent any influence on the predictions, each reference structure was removed from its respective template dataset.

### Preparation of inputs

The sequences of the canonical benchmark set were aligned based on the paired pre- and postfusion structures from their respective PDB files, and any tags or signal peptides were removed. Similarly, the sequences of the real-world benchmark set were processed to remove signal peptides, if present. To ensure consistent comparison with the experimentally determined structures, the sequences used for predictions were delivered directly from the corresponding PDB reference entries. As a result, the transmembrane and cytoplasmic regions were only fully or partially present in a limited number of sequences included in the study. In the cropped-sequence approach, the sequences were cropped into GP2 for both the canonical and real-world benchmark sets (S4 and S5 Table). All template and reference PDB files were cleaned with clean_pdb.py from ions. In a limited number of paired pre- and postfusion structures, poorly aligned terminal regions were cropped prior to prediction to ensure consistent sequence alignment between the two conformational states. These regions did not affect TM-score calculations, as US-align evaluates only structurally matching regions between compared models.

### Structure prediction

All arguments of each run for both cases were the same. ColabFold v1.5.2 used AlphaFold v2.3.1, and --alphafold2_multimer_v3 option was applied for the prediction. In all samples, 5 recycles were utilized and approximately 27–40 models were generated with --save_recycles option. In terms of MSA depth, the default features were applied.

### Scoring and conformational analysis

In conformational analysis, the predicted structures were compared with their respective reference structures based on TM-score (Template Modelling score) to evaluate structural similarity between two protein structures [63]. TM-score analysis was performed using the US-align tool, which identifies optimized structural alignments between compared models and calculates TM-scores based on aligned residues [76]. The predicted models were compared with both the prefusion and postfusion reference structures. The structures in Table 1 were used as reference structures for the canonical benchmark set since they had at least one pair of structures in the PDB with pre- and postfusion states. For the GP sequences of the real-world benchmark set, the LASV GPC structures were chosen as the reference due to their compatibility for comparing pre- and postfusion structures, their structural similarity, and the absence of experimentally determined postfusion structures. Additionally, the original prefusion state structures from the real-world benchmark set were used in the TM-score analyses to obtain precise results (S6 Table). For all models, the pLDDT scores were reported by AlphaFold were used as model confidence estimates. To enable direct comparison with the TM-scores, pLDDT values were normalized to a 0–1 scale by division by 100. The RMSD calculations for the JUNV structures were performed in the ChimeraX based on Cα atoms. For these RMSD analyses and the corresponding structural visualizations, transmembrane and cytoplasmic regions were omitted to ensure consistent structural comparison.

### Analyzing results

The distribution and pLDDT graphs of the TM-scores based on the prefusion and postfusion structures were prepared with R-studio [77,78]. The figures of protein structures were generated with ChimeraX [79,80].

## Supporting information

S1 Table Input sequences of the canonical benchmark set designs. (PDF)

S2 Table Input sequences of the real-world benchmark set designs. Protein sequences were retrieved from the NCBI Protein database. The NCBI Protein accession numbers and UniProtKB accession numbers are provided in the table.
(PDF)

S3 Table Template benchmark set. Structures were obtained from the Protein Data Bank (PDB).
(PDF)

S4 Table Input sequences of GP2 for the canonical benchmark set designs. (PDF)

S5 Table Input sequences of GP2 for the real-world benchmark set designs. (PDF)

S6 Table Reference structures of the real-world benchmark set for the prefusion specific analyses. Structures were obtained from the Protein Data Bank (PDB).
(PDF)

S1 Fig Workflow of the full-length sequence approach for default, all-templates, prefusion-templates and postfusion-templates strategies. (TIF)

S2 Fig Workflow of the cropped sequence approach for default, all-templates, prefusion-templates and postfusion-templates strategies. (TIF)

S3 Fig Predicted models with the highest TM-scores from the real-world benchmark set obtained using the default and postfusion-template strategies within the cropped-sequence approach. TM-score analyses for the postfusion states were performed using the corresponding experimentally determined postfusion structures as references. The models with the highest TM-scores are shown aligned with their respective postfusion reference structures, displayed in grey (respective PDB IDs as following: HA: 1HTM, MARV: 4G2K, LASV1: 5OMI, LASV2: 6JGY), while the predicted models are shown in transparent colors.
(TIF)

S4 Fig Normalized pLDDT and TM-score analyses based on the prefusion conformation of the real-world benchmark set, according to the four template strategies of the cropped sequence approach. TM-score analyses for the prefusion states were conducted using their respective experimentally determined prefusion structures, except for the CHAV analysis, which utilized the JUNV structure due to the absence of an experimentally determined prefusion state for the CHAV. TM-score >=0.45 signifies similar overall structural topology. pLDDT score is considered as; very low, 0–50; low, 50–70; high, 70–90; and very high, 90–100.
(TIF)

S5 Fig Normalized pLDDT and TM-score analyses of the canonical benchmark set, according to the four template strategies of the full-length sequence approach. TM-score >=0.45 signifies similar overall structural topology. pLDDT score is considered as; very low, 0–0.5; low, 0.5–0.7; high, 0.7–0.9; and very high, 0.9–1.
(TIF)

S6 Fig Normalized pLDDT and TM-score analyses of the canonical benchmark set, according to the four template strategies of the cropped sequence approach. TM-score >=0.45 signifies similar overall structural topology. pLDDT score is considered as; very low, 0–0.5; low, 0.5–0.7; high, 0.7–0.9; and very high, 0.9–1.
(TIF)

S7 Fig Normalized pLDDT and TM-score analyses based on the LASV GPC of the real-world benchmark set, according to the four template strategies of the full-length sequence approach. TM-score analyses for pre- and postfusion states were conducted using the LASV GPC pre- and postfusion structure, as experimentally determined postfusion structures are not available for the GP of all viruses. TM-score >=0.45 signifies similar overall structural topology. pLDDT score is considered as; very low, 0–0.5; low, 0.5–0.7; high, 0.7–0.9; and very high, 0.9–1.
(TIF)

S8 Fig Normalized pLDDT and TM-score analyses based on the LASV GPC of the real-world benchmark set, according to the four template strategies of the cropped sequence approach. TM-score analyses for pre- and postfusion states were conducted using the LASV GPC pre- and postfusion structure, as experimentally determined postfusion structures are not available for the GP of all viruses. TM-score >=0.45 signifies similar overall structural topology. pLDDT score is considered as; very low, 0–0.5; low, 0.5–0.7; high, 0.7–0.9; and very high, 0.9–1.
(TIF)
