# Supplementary material for: Prediction of pre- and postfusion conformations of class I fusion proteins with AlphaFold2
Source: PLoS One. 2026 Jun 16;21(6):e0351662. doi: 10.1371/journal.pone.0351662 (PMC13271458; doi:10.1371/journal.pone.0351662)
Supplement: S1 Table — (PDF) [file pone.0351662.s001.pdf]

S1 Table. Input sequences of the canonical benchmark set designs.

> EBOV_3CSY_Zaire-1976
YPYDVPDYAIEGRGARSIPLGVIHNSVLQVSDVDKLVCRDKLSSTNQLRSVGLNLEGNGVATDVPSATK
RWGFRSGVPPKVVNYEAGEWAENCYNLEIKKPDGSECLPAAPDGIRGFPRCRYVHKVSGTGPCAGDFA
FHKEGAFFLYDRLASTVIYRGTTFAEGVVAFLILPQAKKDFFSSHPLREPVNATEDPSSGYYSTTIRYQAT
GFGTNEVEYLFEVDNLTYVQLESRFTPQFLLQLNETIYTSGKRSNTTGKLIWKVNPEIDTTIGEWAFWET
KKNLTRKIRSEELSFTVVTHHQDTGEESASSGKLGLITNTIAGVAGLITGGRRTRR:
YPYDVPDYAIEGRGARSIPLGVIHNSVLQVSDVDKLVCRDKLSSTNQLRSVGLNLEGNGVATDVPSATK
RWGFRSGVPPKVVNYEAGEWAENCYNLEIKKPDGSECLPAAPDGIRGFPRCRYVHKVSGTGPCAGDFA
FHKEGAFFLYDRLASTVIYRGTTFAEGVVAFLILPQAKKDFFSSHPLREPVNATEDPSSGYYSTTIRYQAT
GFGTNEVEYLFEVDNLTYVQLESRFTPQFLLQLNETIYTSGKRSNTTGKLIWKVNPEIDTTIGEWAFWET
KKNLTRKIRSEELSFTVVTHHQDTGEESASSGKLGLITNTIAGVAGLITGGRRTRR:
YPYDVPDYAIEGRGARSIPLGVIHNSVLQVSDVDKLVCRDKLSSTNQLRSVGLNLEGNGVATDVPSATK
RWGFRSGVPPKVVNYEAGEWAENCYNLEIKKPDGSECLPAAPDGIRGFPRCRYVHKVSGTGPCAGDFA
FHKEGAFFLYDRLASTVIYRGTTFAEGVVAFLILPQAKKDFFSSHPLREPVNATEDPSSGYYSTTIRYQAT
GFGTNEVEYLFEVDNLTYVQLESRFTPQFLLQLNETIYTSGKRSNTTGKLIWKVNPEIDTTIGEWAFWET
KKNLTRKIRSEELSFTVVTHHQDTGEESASSGKLGLITNTIAGVAGLITGGRRTRR:
EAIVNAQPKCNPNLHYWTTQDEGAAIGLAWIPYFGPAAEGIYTEGLMHNQDGLICGLRQLANETTQALQ
LFLRATTELRTFSILNRKAIDFLLQRWGGTCHILGPDCCIEPHDWTKNITDKIDQIIHDFVD:
EAIVNAQPKCNPNLHYWTTQDEGAAIGLAWIPYFGPAAEGIYTEGLMHNQDGLICGLRQLANETTQALQ
LFLRATTELRTFSILNRKAIDFLLQRWGGTCHILGPDCCIEPHDWTKNITDKIDQIIHDFVD:
EAIVNAQPKCNPNLHYWTTQDEGAAIGLAWIPYFGPAAEGIYTEGLMHNQDGLICGLRQLANETTQALQ
LFLRATTELRTFSILNRKAIDFLLQRWGGTCHILGPDCCIEPHDWTKNITDKIDQIIHDFVD

> HA_A_Aichi_2_1968_H3N2
QDLPGNDNSTATLCLGHHAVPNGTLVKTITDDQIEVTNATELVQSSSTGKICNNPHRILDGIDCTLIDALL
GDPHCDVFQNETWDLFVERSKAFSNCYPYDVPDYASLRSLVASSGTLEFITEGFTWTGVTQNGGSNACK
RGPGSGFFSRLNWLTKSGSTYPVLNVTMPNNDNFDKLYIWGIHHPSTNQEQTSLYVQASGRVTVSTRRS
QQTIIPNIGSRPWVRGLSSRISIYWTIVKPGDVLVINSNGNLIAPRGYFKMRTGKSSIMRSDAPIDTCISECIT
PNGSIPNDKPFQNVNKITYGACPKYVKQNTLKLATGMRNVPEKQT:
QDLPGNDNSTATLCLGHHAVPNGTLVKTITDDQIEVTNATELVQSSSTGKICNNPHRILDGIDCTLIDALL
GDPHCDVFQNETWDLFVERSKAFSNCYPYDVPDYASLRSLVASSGTLEFITEGFTWTGVTQNGGSNACK
RGPGSGFFSRLNWLTKSGSTYPVLNVTMPNNDNFDKLYIWGIHHPSTNQEQTSLYVQASGRVTVSTRRS
QQTIIPNIGSRPWVRGLSSRISIYWTIVKPGDVLVINSNGNLIAPRGYFKMRTGKSSIMRSDAPIDTCISECIT
PNGSIPNDKPFQNVNKITYGACPKYVKQNTLKLATGMRNVPEKQT:
QDLPGNDNSTATLCLGHHAVPNGTLVKTITDDQIEVTNATELVQSSSTGKICNNPHRILDGIDCTLIDALL
GDPHCDVFQNETWDLFVERSKAFSNCYPYDVPDYASLRSLVASSGTLEFITEGFTWTGVTQNGGSNACK
RGPGSGFFSRLNWLTKSGSTYPVLNVTMPNNDNFDKLYIWGIHHPSTNQEQTSLYVQASGRVTVSTRRS
QQTIIPNIGSRPWVRGLSSRISIYWTIVKPGDVLVINSNGNLIAPRGYFKMRTGKSSIMRSDAPIDTCISECIT
PNGSIPNDKPFQNVNKITYGACPKYVKQNTLKLATGMRNVPEKQT:
GLFGAIAGFIENGWEGMIDGWYGFRHQNSEGTGQAADLKSTQAAIDQINGKLNRVIEKTNEKFHQIEKE
FSEVEGRIQDLEKYVEDTKIDLWSYNAELLVALENQHTIDLTDSEMNKLFEKTRRQLRENAEEMGNGCF
KIYHKCDNACIESIRNGTYDHDVYRDEALNNRFQIKG:
GLFGAIAGFIENGWEGMIDGWYGFRHQNSEGTGQAADLKSTQAAIDQINGKLNRVIEKTNEKFHQIEKE
FSEVEGRIQDLEKYVEDTKIDLWSYNAELLVALENQHTIDLTDSEMNKLFEKTRRQLRENAEEMGNGCF
KIYHKCDNACIESIRNGTYDHDVYRDEALNNRFQIKG:
GLFGAIAGFIENGWEGMIDGWYGFRHQNSEGTGQAADLKSTQAAIDQINGKLNRVIEKTNEKFHQIEKE
FSEVEGRIQDLEKYVEDTKIDLWSYNAELLVALENQHTIDLTDSEMNKLFEKTRRQLRENAEEMGNGCF
KIYHKCDNACIESIRNGTYDHDVYRDEALNNRFQIKG

> LASV1_Josiah_1976
MGQIVTFFQEVPHVIEEVMNIVLIALSVLAVLKGLYNFATCGLVGLVTFLLLCGRSCTTSLYKGVYELQT
LELNMETLNMTMPLSCTKNNSHHYIMVGNETGLELTLTNTSIINHKFCNLSDAHKKNLYDHALMSIISTF

HLSIPNFNQYEAMSCDFNGGKISVQYNLSHSYAGDAANHCGTVANGVLQTFMRMAWGGSYIALDSGR
GNWDCIMTSYQYLIIQNTTWEDHCQFSRPSPIGYLGLLSQRTRDIYISRRLL:
MGQIVTFFQEVPHVIEEVMNIVLIALSVLAVLKGLYNFATCGLVGLVTFLLLCGRSCTTSLYKGVYELQT
LELNMETLNMTMPLSCTKNNSHHYIMVGNETGLELTLTNTSIINHKFCNLSDAHKKNLYDHALMSIISTF
HLSIPNFNQYEAMSCDFNGGKISVQYNLSHSYAGDAANHCGTVANGVLQTFMRMAWGGSYIALDSGR
GNWDCIMTSYQYLIIQNTTWEDHCQFSRPSPIGYLGLLSQRTRDIYISRRLL:
MGQIVTFFQEVPHVIEEVMNIVLIALSVLAVLKGLYNFATCGLVGLVTFLLLCGRSCTTSLYKGVYELQT
LELNMETLNMTMPLSCTKNNSHHYIMVGNETGLELTLTNTSIINHKFCNLSDAHKKNLYDHALMSIISTF
HLSIPNFNQYEAMSCDFNGGKISVQYNLSHSYAGDAANHCGTVANGVLQTFMRMAWGGSYIALDSGR
GNWDCIMTSYQYLIIQNTTWEDHCQFSRPSPIGYLGLLSQRTRDIYISRRLL:
GTFTWTLSDSEGKDTPGGYCLTRWMLIEAELKCFGNTAVAKCNEKHDEEFCDMLRLFDFNKQAIQRLK
AEAQMSIQLINKAVNALINDQLIMKNHLRDIMGIPYCNYSKYWYLNHTTTGRTSLPKCWLVSNGSYLNE
THFSDDIEQQADNMITEMLQKEYMERQGKTPLGLVDLFVFSTSFYLISIFLHLVKIPTHRHIVGKSCPKPH
RLNHMGICSCGLYKQPGVPVKWKR:
GTFTWTLSDSEGKDTPGGYCLTRWMLIEAELKCFGNTAVAKCNEKHDEEFCDMLRLFDFNKQAIQRLK
AEAQMSIQLINKAVNALINDQLIMKNHLRDIMGIPYCNYSKYWYLNHTTTGRTSLPKCWLVSNGSYLNE
THFSDDIEQQADNMITEMLQKEYMERQGKTPLGLVDLFVFSTSFYLISIFLHLVKIPTHRHIVGKSCPKPH
RLNHMGICSCGLYKQPGVPVKWKR:
GTFTWTLSDSEGKDTPGGYCLTRWMLIEAELKCFGNTAVAKCNEKHDEEFCDMLRLFDFNKQAIQRLK
AEAQMSIQLINKAVNALINDQLIMKNHLRDIMGIPYCNYSKYWYLNHTTTGRTSLPKCWLVSNGSYLNE
THFSDDIEQQADNMITEMLQKEYMERQGKTPLGLVDLFVFSTSFYLISIFLHLVKIPTHRHIVGKSCPKPH
RLNHMGICSCGLYKQPGVPVKWKR

> LASV2_Josiah_1976
MGQIVTFFQEVPHVIEEVMNIVLIALSVLAVLKGLYNFATCGLVGLVTFLLLCGRSCTTSLYKGVYELQT
LELNMETLNMTMPLSCTKNNSHHYIMVGNETGLELTLTNTSIINHKFCNLSDAHKKNLYDHALMSIISTF
HLSIPNFNQYEAMSCDFNGGKISVQYNLSHSYAGDAANHCGTVANGVLQTFMRMAWGGSYIALDSGC
GNWDCIMTSYQYLIIQNTTWEDHCQFSRPSPIGYLGLLSQRTRDIYISRRRR:
MGQIVTFFQEVPHVIEEVMNIVLIALSVLAVLKGLYNFATCGLVGLVTFLLLCGRSCTTSLYKGVYELQT
LELNMETLNMTMPLSCTKNNSHHYIMVGNETGLELTLTNTSIINHKFCNLSDAHKKNLYDHALMSIISTF
HLSIPNFNQYEAMSCDFNGGKISVQYNLSHSYAGDAANHCGTVANGVLQTFMRMAWGGSYIALDSGC
GNWDCIMTSYQYLIIQNTTWEDHCQFSRPSPIGYLGLLSQRTRDIYISRRRR:
MGQIVTFFQEVPHVIEEVMNIVLIALSVLAVLKGLYNFATCGLVGLVTFLLLCGRSCTTSLYKGVYELQT
LELNMETLNMTMPLSCTKNNSHHYIMVGNETGLELTLTNTSIINHKFCNLSDAHKKNLYDHALMSIISTF
HLSIPNFNQYEAMSCDFNGGKISVQYNLSHSYAGDAANHCGTVANGVLQTFMRMAWGGSYIALDSGC
GNWDCIMTSYQYLIIQNTTWEDHCQFSRPSPIGYLGLLSQRTRDIYISRRRR:
GTFTWTLSDSEGKDTPGGYCLTRWMLIEAELKCFGNTAVAKCNEKHDEEFCDMLRLFDFNKQAIQRLK
APAQTSIQLINKAVNALINDQLIMKNHLRDIMCIPYCNYSKYWYLNHTTTGRTSLPKCWLVSNGSYLNE
THFSDDIEQQADNMITEMLQKEYMERQGKTPLGLVD:
GTFTWTLSDSEGKDTPGGYCLTRWMLIEAELKCFGNTAVAKCNEKHDEEFCDMLRLFDFNKQAIQRLK
APAQTSIQLINKAVNALINDQLIMKNHLRDIMCIPYCNYSKYWYLNHTTTGRTSLPKCWLVSNGSYLNE
THFSDDIEQQADNMITEMLQKEYMERQGKTPLGLVD:
GTFTWTLSDSEGKDTPGGYCLTRWMLIEAELKCFGNTAVAKCNEKHDEEFCDMLRLFDFNKQAIQRLK
APAQTSIQLINKAVNALINDQLIMKNHLRDIMCIPYCNYSKYWYLNHTTTGRTSLPKCWLVSNGSYLNE
THFSDDIEQQADNMITEMLQKEYMERQGKTPLGLVD

> MARV_6BP2_RAVN-87
KTLPVLEIASNSQPQDVDSVCSGTLQKTEDVHLMGFTLSGQKVADSPLEASKRWAFRTGVPPKNVEYTE
GEEAKTCYNISVTDPSGKSLLLDPPSNIRDYPKCKTVHHIQGQNPHAQGIALHLWGAFFLYDRVASTTM
YRGKVFTEGNIAAMIVNKTVHRMIFSRQGQGYRHMNLTSTNKYWTSSNETQRNDTGCFGILQEYNSTN
NQTCPPSLKPPSLPTVTPSIHSTNTQINTAKSGTRPPIYFRKKR:
KTLPVLEIASNSQPQDVDSVCSGTLQKTEDVHLMGFTLSGQKVADSPLEASKRWAFRTGVPPKNVEYTE
GEEAKTCYNISVTDPSGKSLLLDPPSNIRDYPKCKTVHHIQGQNPHAQGIALHLWGAFFLYDRVASTTM
YRGKVFTEGNIAAMIVNKTVHRMIFSRQGQGYRHMNLTSTNKYWTSSNETQRNDTGCFGILQEYNSTN
NQTCPPSLKPPSLPTVTPSIHSTNTQINTAKSGTRPPIYFRKKR:

KTLPVLEIASNSQPQDVDSVCSGTLQKTEDVHLMGFTLSGQKVADSPLEASKRWAFRTGVPPKNVEYTE
GEEAKTCYNISVTDPSGKSLLLDPPSNIRDYPKCKTVHHIQGQNPHAQGIALHLWGAFFLYDRVASTTM
YRGKVFTEGNIAAMIVNKTVHRMIFSRQGQGYRHMNLTSTNKYWTSSNETQRNDTGCFGILQEYNSTN
NQTCPPSLKPPSLPTVTPSIHSTNTQINTAKSGTRPPIYFRKKR:
SILAKEGDIGPNLDGLINTEIDFDPIPNTETIFDESPSFNTSTNEEQHTPPNISLTFSYFPDKNGDTAYSGENE
NDCDAELRIWSVQEDDLAAGLSWIPFFGPGIEGLYTAGLIKNQNNLVCRLRRLANQTAKSLELLLRVTTE
ERTFSLINRHAIDFLLTRWGGTCKVLGPDCCIGIEDLSKNISEQIDKIRKDEQKEETG:
SILAKEGDIGPNLDGLINTEIDFDPIPNTETIFDESPSFNTSTNEEQHTPPNISLTFSYFPDKNGDTAYSGENE
NDCDAELRIWSVQEDDLAAGLSWIPFFGPGIEGLYTAGLIKNQNNLVCRLRRLANQTAKSLELLLRVTTE
ERTFSLINRHAIDFLLTRWGGTCKVLGPDCCIGIEDLSKNISEQIDKIRKDEQKEETG:
SILAKEGDIGPNLDGLINTEIDFDPIPNTETIFDESPSFNTSTNEEQHTPPNISLTFSYFPDKNGDTAYSGENE
NDCDAELRIWSVQEDDLAAGLSWIPFFGPGIEGLYTAGLIKNQNNLVCRLRRLANQTAKSLELLLRVTTE
ERTFSLINRHAIDFLLTRWGGTCKVLGPDCCIGIEDLSKNISEQIDKIRKDEQKEETG

> RSV_strainA2_4MMS_3RKI
QNITEEFYQSTCSAVSKGYLSALRTGWYTSVITIELSNIKENKCNGTDAKVKLIKQELDKYKNAVTELQL
LMQSTPATNNRA:
QNITEEFYQSTCSAVSKGYLSALRTGWYTSVITIELSNIKENKCNGTDAKVKLIKQELDKYKNAVTELQL
LMQSTPATNNRA:
QNITEEFYQSTCSAVSKGYLSALRTGWYTSVITIELSNIKENKCNGTDAKVKLIKQELDKYKNAVTELQL
LMQSTPATNNRA:
FLGFLLGVGSAIASGVAVSKVLHLEGEVNKIKSALLSTNKAVVSLSNGVSVLTFKVLDLKNYIDKQLLPI
LNKQSCSISNIETVIEFQQKNNRLLEITREFSVNAGVTTPVSTYMLTNSELLSLINDMPITNDQKKLMSNN
VQIVRQQSYSIMSIIKEEVLAYVVQLPLYGVIDTPCWKLHTSPLCTTNTKEGSNICLTRTDRGWYCDNAG
SVSFFPQAETCKVQSNRVFCDTMNSLTLPSEVNLCNVDIFNPKYDCKIMTSKTDVSSSVITSLGAIVSCYG
KTKCTASNKNRGIIKTFSNGCDYVSNKGVDTVSVGNTLYYVNKQEGKSLYVKGEPIINFYDPLVFPSDEF
DASISQVNEKINQSLAFIRKSDELLSAIG:
FLGFLLGVGSAIASGVAVSKVLHLEGEVNKIKSALLSTNKAVVSLSNGVSVLTFKVLDLKNYIDKQLLPI
LNKQSCSISNIETVIEFQQKNNRLLEITREFSVNAGVTTPVSTYMLTNSELLSLINDMPITNDQKKLMSNN
VQIVRQQSYSIMSIIKEEVLAYVVQLPLYGVIDTPCWKLHTSPLCTTNTKEGSNICLTRTDRGWYCDNAG
SVSFFPQAETCKVQSNRVFCDTMNSLTLPSEVNLCNVDIFNPKYDCKIMTSKTDVSSSVITSLGAIVSCYG
KTKCTASNKNRGIIKTFSNGCDYVSNKGVDTVSVGNTLYYVNKQEGKSLYVKGEPIINFYDPLVFPSDEF
DASISQVNEKINQSLAFIRKSDELLSAIG:
FLGFLLGVGSAIASGVAVSKVLHLEGEVNKIKSALLSTNKAVVSLSNGVSVLTFKVLDLKNYIDKQLLPI
LNKQSCSISNIETVIEFQQKNNRLLEITREFSVNAGVTTPVSTYMLTNSELLSLINDMPITNDQKKLMSNN
VQIVRQQSYSIMSIIKEEVLAYVVQLPLYGVIDTPCWKLHTSPLCTTNTKEGSNICLTRTDRGWYCDNAG
SVSFFPQAETCKVQSNRVFCDTMNSLTLPSEVNLCNVDIFNPKYDCKIMTSKTDVSSSVITSLGAIVSCYG
KTKCTASNKNRGIIKTFSNGCDYVSNKGVDTVSVGNTLYYVNKQEGKSLYVKGEPIINFYDPLVFPSDEF
DASISQVNEKINQSLAFIRKSDELLSAIG
