# Supplementary material for: Prediction of pre- and postfusion conformations of class I fusion proteins with AlphaFold2
Source: PLoS One. 2026 Jun 16;21(6):e0351662. doi: 10.1371/journal.pone.0351662 (PMC13271458; doi:10.1371/journal.pone.0351662)
Supplement: S2 Table — Protein sequences were retrieved from the NCBI Protein database. The NCBI Protein accession numbers and UniProtKB accession numbers are provided in the table. (PDF) [file pone.0351662.s002.pdf]

S2 Table. Input sequences of the real-world benchmark set designs. Protein sequences were retrieved from the NCBI Protein database (1). The NCBI Protein accession numbers and UniProtKB accession numbers are provided in the table (2).

> JUNV_NP899218 | NCBI Protein: NP_899218.1 | UniProtKB: Q6UY73 | Junín virus<br>MGQFISFMQEIPTFLQEALNIALVAVSLIAIIKGIVNLYKSGLFQFFVFLALAGRSCTEEAFKIGLHTEFQTV<br>SFSMVGLFSNNPHDLPLLCTLNKSHLYIKGGNASFQISFDDIAVLLPQYDVIIQHPADMSWCSKSDDQIW<br>LSQWFMNAVGHDWHLDPPFLCRNRTKTEGFIFQVNTSKTGVNENYAKKFKTGMHHLYREYPDSCPNG<br>KLCLMKAQPTSWPLQCPLDHVNTLHFLTRGKNIQLPRRSLKAFFSWSLTDSSGKDTPGGYCLEEWMLV<br>AAKMKCFGNTAVAKCNLNHDSEFCDMLRLFDYNKNAIKTLNDETKKQVNLMGQTINALISDNLLMKN<br>KIRELMSVPYCNYTKFWYVNHTLSGQHSLPRCWLIKNNSYLNISDFRNDWILESDFLISEMLSKEYSDRQ<br>GKTPLTLVDICFWSTVFFTASLFLHLVGIPTHRHIRGEACPLPHRLNSLGGCRCGKYPNLKKPTVWRRGH:<br>MGQFISFMQEIPTFLQEALNIALVAVSLIAIIKGIVNLYKSGLFQFFVFLALAGRSCTEEAFKIGLHTEFQTV<br>SFSMVGLFSNNPHDLPLLCTLNKSHLYIKGGNASFQISFDDIAVLLPQYDVIIQHPADMSWCSKSDDQIW<br>LSQWFMNAVGHDWHLDPPFLCRNRTKTEGFIFQVNTSKTGVNENYAKKFKTGMHHLYREYPDSCPNG<br>KLCLMKAQPTSWPLQCPLDHVNTLHFLTRGKNIQLPRRSLKAFFSWSLTDSSGKDTPGGYCLEEWMLV<br>AAKMKCFGNTAVAKCNLNHDSEFCDMLRLFDYNKNAIKTLNDETKKQVNLMGQTINALISDNLLMKN<br>KIRELMSVPYCNYTKFWYVNHTLSGQHSLPRCWLIKNNSYLNISDFRNDWILESDFLISEMLSKEYSDRQ<br>GKTPLTLVDICFWSTVFFTASLFLHLVGIPTHRHIRGEACPLPHRLNSLGGCRCGKYPNLKKPTVWRRGH:<br>MGQFISFMQEIPTFLQEALNIALVAVSLIAIIKGIVNLYKSGLFQFFVFLALAGRSCTEEAFKIGLHTEFQTV<br>SFSMVGLFSNNPHDLPLLCTLNKSHLYIKGGNASFQISFDDIAVLLPQYDVIIQHPADMSWCSKSDDQIW<br>LSQWFMNAVGHDWHLDPPFLCRNRTKTEGFIFQVNTSKTGVNENYAKKFKTGMHHLYREYPDSCPNG<br>KLCLMKAQPTSWPLQCPLDHVNTLHFLTRGKNIQLPRRSLKAFFSWSLTDSSGKDTPGGYCLEEWMLV<br>AAKMKCFGNTAVAKCNLNHDSEFCDMLRLFDYNKNAIKTLNDETKKQVNLMGQTINALISDNLLMKN<br>KIRELMSVPYCNYTKFWYVNHTLSGQHSLPRCWLIKNNSYLNISDFRNDWILESDFLISEMLSKEYSDRQ<br>GKTPLTLVDICFWSTVFFTASLFLHLVGIPTHRHIRGEACPLPHRLNSLGGCRCGKYPNLKKPTVWRRGH
> MACV_NP899212 | NCBI Protein: NP_899212.1 | UniProtKB: Q8AZ57 | Machupo virus<br>MGQLISFFQEIPVFLQEALNIALVAVSLIAVIKGIINLYKSGLFQFIFFLLLAGRSCSDGTFKIGLHTEFQSVT<br>LTMQRLLANHSNELPSLCMLNNSFYYMRGGVNTFLIRVSDISVLMKEYDVSIYEPEDLGNCLNKSDSSW<br>AIHWFSNALGHDWLMDPPMLCRNKTKKEGSNIQFNISKADDARVYGKKIRNGMRHLFRGFHDPCEEGK<br>VCYLTINQCGDPSSFDYCGVNHLSKCQFDHVNTLHFLVRSKTHLNFERSLKAFFSWSLTDSSGKDMPGG<br>YCLEEWMLIAAKMKCFGNTAVAKCNQNHDSEFCDMLRLFDYNKNAIKTLNDESKKEINLLSQTVNALI<br>SDNLLMKNKIKELMSIPYCNYTKFWYVNHTLTGQHTLPRCWLIRNGSYLNTSEFRNDWILESDHLISEM<br>LSKEYAERQGKTPITLVDICFWSTIFFTASLFLHLVGIPTHRHLKGEACPLPHKLDSFGGCRCGKYPRLKK<br>PTIWHKRH:<br>MGQLISFFQEIPVFLQEALNIALVAVSLIAVIKGIINLYKSGLFQFIFFLLLAGRSCSDGTFKIGLHTEFQSVT<br>LTMQRLLANHSNELPSLCMLNNSFYYMRGGVNTFLIRVSDISVLMKEYDVSIYEPEDLGNCLNKSDSSW<br>AIHWFSNALGHDWLMDPPMLCRNKTKKEGSNIQFNISKADDARVYGKKIRNGMRHLFRGFHDPCEEGK<br>VCYLTINQCGDPSSFDYCGVNHLSKCQFDHVNTLHFLVRSKTHLNFERSLKAFFSWSLTDSSGKDMPGG<br>YCLEEWMLIAAKMKCFGNTAVAKCNQNHDSEFCDMLRLFDYNKNAIKTLNDESKKEINLLSQTVNALI<br>SDNLLMKNKIKELMSIPYCNYTKFWYVNHTLTGQHTLPRCWLIRNGSYLNTSEFRNDWILESDHLISEM<br>LSKEYAERQGKTPITLVDICFWSTIFFTASLFLHLVGIPTHRHLKGEACPLPHKLDSFGGCRCGKYPRLKK<br>PTIWHKRH:<br>MGQLISFFQEIPVFLQEALNIALVAVSLIAVIKGIINLYKSGLFQFIFFLLLAGRSCSDGTFKIGLHTEFQSVT<br>LTMQRLLANHSNELPSLCMLNNSFYYMRGGVNTFLIRVSDISVLMKEYDVSIYEPEDLGNCLNKSDSSW<br>AIHWFSNALGHDWLMDPPMLCRNKTKKEGSNIQFNISKADDARVYGKKIRNGMRHLFRGFHDPCEEGK<br>VCYLTINQCGDPSSFDYCGVNHLSKCQFDHVNTLHFLVRSKTHLNFERSLKAFFSWSLTDSSGKDMPGG<br>YCLEEWMLIAAKMKCFGNTAVAKCNQNHDSEFCDMLRLFDYNKNAIKTLNDESKKEINLLSQTVNALI<br>SDNLLMKNKIKELMSIPYCNYTKFWYVNHTLTGQHTLPRCWLIRNGSYLNTSEFRNDWILESDHLISEM<br>LSKEYAERQGKTPITLVDICFWSTIFFTASLFLHLVGIPTHRHLKGEACPLPHKLDSFGGCRCGKYPRLKK<br>PTIWHKRH

> CHAVB-2003 | NCBI Protein: YP_001816782.1 | UniProtKB: B2C4J0 | Chapare virus
GLVNLWKSGLFQLLVFLILAGRSCSFKIGRSTELQNITINMLKVFEDHPISCTVNKTLYYIRESENATWCV
EIAALDMSVLLSPHDPRVMGNLSNCVHPDIKHRSELLGLLEWILRALKYDFLNYPPLLCEKVTSSVNETR
IQINVSDSAGSHDFKETMLQRLAILFGTKLMFDKTPKQFIVIRNQTWVNQCKSNHVNTLHLMMANAGH
AVKLRRLQGVFTWTITDAAGNDMPGGYCLERWMLVTSDLKCFGNTALAKCNLNHDSEFCDMLKLFEF
NKKAIESLNDNTKNKVNLLTHSINALISDNLLMKNRLKELLDTPYCNYTKFWYVNHTITGEHSLPRCWM
VKNNSYLNESEFRNDWILESDHLLSEMLNKEYFDRQGKTPITLVD:
GLVNLWKSGLFQLLVFLILAGRSCSFKIGRSTELQNITINMLKVFEDHPISCTVNKTLYYIRESENATWCV
EIAALDMSVLLSPHDPRVMGNLSNCVHPDIKHRSELLGLLEWILRALKYDFLNYPPLLCEKVTSSVNETR
IQINVSDSAGSHDFKETMLQRLAILFGTKLMFDKTPKQFIVIRNQTWVNQCKSNHVNTLHLMMANAGH
AVKLRRLQGVFTWTITDAAGNDMPGGYCLERWMLVTSDLKCFGNTALAKCNLNHDSEFCDMLKLFEF
NKKAIESLNDNTKNKVNLLTHSINALISDNLLMKNRLKELLDTPYCNYTKFWYVNHTITGEHSLPRCWM
VKNNSYLNESEFRNDWILESDHLLSEMLNKEYFDRQGKTPITLVD:
GLVNLWKSGLFQLLVFLILAGRSCSFKIGRSTELQNITINMLKVFEDHPISCTVNKTLYYIRESENATWCV
EIAALDMSVLLSPHDPRVMGNLSNCVHPDIKHRSELLGLLEWILRALKYDFLNYPPLLCEKVTSSVNETR
IQINVSDSAGSHDFKETMLQRLAILFGTKLMFDKTPKQFIVIRNQTWVNQCKSNHVNTLHLMMANAGH
AVKLRRLQGVFTWTITDAAGNDMPGGYCLERWMLVTSDLKCFGNTALAKCNLNHDSEFCDMLKLFEF
NKKAIESLNDNTKNKVNLLTHSINALISDNLLMKNRLKELLDTPYCNYTKFWYVNHTITGEHSLPRCWM
VKNNSYLNESEFRNDWILESDHLLSEMLNKEYFDRQGKTPITLVD

> LUJV | NCBI Protein: ACR56359.1 | UniProtKB: C5ILC1 | Lujo virus
MGQIVAVFQAIPEILNEAINIVIIVIIMFTLIKGVFNLYKSGLFQLVIFLLLCGKRCDSSLLSGFNLETVHFN
MSLLSSIPMVSEQQHCIQHNHSSITFSLLTNKSDLEKCNFTRLQAVDRVIFDLFREFHHRVGDFPVTSDLK
CSHNTSYRVIEYEVTKESLPRLQEAVSTLFPDLHLSEDRFLQIQAHDDKNCTGLHPLNYLRLLKENSETH
YKVRKLMKLFQWSLSDETGSPLPGGHCLERWLIFASDIKCFDNAAIAKCNKEHDEEFCDMLRLFDYNKA
SIAKLRGEASSSINLLSGRINAIISDTLLMRSSLKRLMGIPYCNYTKFWYLNHTKLGIHSLPRCWLVSNGSY
LNETKFTHDMEDEADKLLTEMLKKEYVRRQEKTPITLMDILMFSVSFYMFSVTLCICNIPTHRHITGLPCP
KPHRLRKNGTCACGFFKSINRSTGWAKH:
MGQIVAVFQAIPEILNEAINIVIIVIIMFTLIKGVFNLYKSGLFQLVIFLLLCGKRCDSSLLSGFNLETVHFN
MSLLSSIPMVSEQQHCIQHNHSSITFSLLTNKSDLEKCNFTRLQAVDRVIFDLFREFHHRVGDFPVTSDLK
CSHNTSYRVIEYEVTKESLPRLQEAVSTLFPDLHLSEDRFLQIQAHDDKNCTGLHPLNYLRLLKENSETH
YKVRKLMKLFQWSLSDETGSPLPGGHCLERWLIFASDIKCFDNAAIAKCNKEHDEEFCDMLRLFDYNKA
SIAKLRGEASSSINLLSGRINAIISDTLLMRSSLKRLMGIPYCNYTKFWYLNHTKLGIHSLPRCWLVSNGSY
LNETKFTHDMEDEADKLLTEMLKKEYVRRQEKTPITLMDILMFSVSFYMFSVTLCICNIPTHRHITGLPCP
KPHRLRKNGTCACGFFKSINRSTGWAKH:
MGQIVAVFQAIPEILNEAINIVIIVIIMFTLIKGVFNLYKSGLFQLVIFLLLCGKRCDSSLLSGFNLETVHFN
MSLLSSIPMVSEQQHCIQHNHSSITFSLLTNKSDLEKCNFTRLQAVDRVIFDLFREFHHRVGDFPVTSDLK
CSHNTSYRVIEYEVTKESLPRLQEAVSTLFPDLHLSEDRFLQIQAHDDKNCTGLHPLNYLRLLKENSETH
YKVRKLMKLFQWSLSDETGSPLPGGHCLERWLIFASDIKCFDNAAIAKCNKEHDEEFCDMLRLFDYNKA
SIAKLRGEASSSINLLSGRINAIISDTLLMRSSLKRLMGIPYCNYTKFWYLNHTKLGIHSLPRCWLVSNGSY
LNETKFTHDMEDEADKLLTEMLKKEYVRRQEKTPITLMDILMFSVSFYMFSVTLCICNIPTHRHITGLPCP
KPHRLRKNGTCACGFFKSINRSTGWAKH

References

1. National Center for Biotechnology Information (NCBI). National Library of Medicine (US), National Center for Biotechnology Information. 2025 [cited 2025 Dec 17]. National Center for Biotechnology Information (NCBI) - Protein. Available from: https://www.ncbi.nlm.nih.gov/protein/

2. Bateman A, Martin MJ, Orchard S, Magrane M, Adesina A, Ahmad S, et al. UniProt: the Universal Protein Knowledgebase in 2025. Nucleic Acids Res. 2025 Jan 6;53(D1):D609–17. doi:10.1093/nar/gkae1010
