# Supplementary material for: Prediction of pre- and postfusion conformations of class I fusion proteins with AlphaFold2
Source: PLoS One. 2026 Jun 16;21(6):e0351662. doi: 10.1371/journal.pone.0351662 (PMC13271458; doi:10.1371/journal.pone.0351662)
Supplement: S3 Table — Structures were obtained from the Protein Data Bank (PDB). (PDF) [file pone.0351662.s003.pdf]

S3 Table. Template benchmark set for designs of both the canonical and real-world benchmark sets. Structures were obtained from the Protein Data Bank (PDB) (1).

VIRUS	PDB	CONFORMATION	PDB DOI	PAPER DOI
EBOV	5F1B	PRE	(2)	(3)
EBOV	5JQ3	PRE	(4)	(5)
EBOV	6G95	PRE	(6)	(7)
EBOV	6HS4	PRE	(8)	(9)
EBOV	6VKM	PRE	(10)	(11)
EBOV	7LYD	PRE	(12)	Not available; associated publication listed as "To be published"
EBOV	7SSQ	PRE	(13)	Not available; associated publication listed as "To be published"
**EBOV**	**2EBO**	**POST**	(14)	(15)
HA	4BGZ	PRE	(16)	(17)
HA	5BR0	PRE	(18)	(19)
HA	6VMZ	PRE	(20)	(21)
**HA**	**1HTM**	**POST**	(22)	(23)
HA	4NKJ	POST	(24)	(25)
HA	6Y5L	POST	(26)	(27)
LASV	7S8H	PRE	(28)	(29)
LASV	7SGD	PRE	(30)	(31)
LASV	7UL7	PRE	(32)	(33)
**LASV**	**5VK2**	**PRE**	(34)	(35)
LASV	8EJD	PRE	(36)	(37)
LASV	8EJF	PRE	(38)	(37)
**LASV**	**5OMI**	**POST**	(39)	(40)
**LASV**	**6JGY**	**POST**	(41)	(42)
MARV	5UQY	PRE	(43)	(44)
**MARV**	**6BP2**	**PRE**	(45)	(46)
**MARV**	**4G2K**	**POST**	(47)	(48)
RSV	5EA8	PRE	(49)	(50)
RSV	5UDE	PRE	(51)	(52)
RSV	5WB0	PRE	(53)	(54)
RSV	6EAD	PRE	(55)	Not available; associated publication listed as "To be published"
**RSV**	**4MMS**	**PRE**	(56)	(57)
**RSV**	**3RKI**	**POST**	(58)	(59)
RSV	3RRR	POST	(60)	(61)

*PRE=Prefusion conformation, POST=Postfusion conformation

**Bold terms were left out when predicting their own virus structure and used only for predicting structures of other viruses.

References

1. Berman HM. The Protein Data Bank. Nucleic Acids Res. 2000 Jan 1;28(1):235–42. doi:10.1093/nar/28.1.235

2. Wang H, Shi Y, Song J, Qi J, Lu G, Yan J, et al. Structural basis of Ebola virus entry: viral glycoprotein bound to its endosomal receptor Niemann-Pick C1. Worldwide Protein Data Bank. 2016. doi:10.2210/pdb5f1b/pdb

3. Wang H, Shi Y, Song J, Qi J, Lu G, Yan J, et al. Ebola Viral Glycoprotein Bound to Its Endosomal Receptor Niemann-Pick C1. Cell. 2016 Jan;164(1–2):258–68. doi:10.1016/j.cell.2015.12.044

4. Zhao Y, Ren J, Stuart DI. Crystal structure of Ebola glycoprotein. Worldwide Protein Data Bank. 2016. doi:10.2210/pdb5jq3/pdb

5. Zhao Y, Ren J, Harlos K, Jones DM, Zeltina A, Bowden TA, et al. Toremifene interacts with and destabilizes the Ebola virus glycoprotein. Nature. 2016 Jul 7;535(7610):169–72. doi:10.1038/nature18615

6. Zhao Y, Ren J, Fry EE, Xiao J, Townsend AR, Stuart DI. Crystal structure of Ebolavirus glycoprotein in complex with thioridazine. Worldwide Protein Data Bank. 2018. doi:10.2210/pdb6g95/pdb

7. Zhao Y, Ren J, Fry EE, Xiao J, Townsend AR, Stuart DI. Structures of Ebola Virus Glycoprotein Complexes with Tricyclic Antidepressant and Antipsychotic Drugs. J Med Chem. 2018 Jun 14;61(11):4938–45. doi:10.1021/acs.jmedchem.8b00350

8. Ren J, Zhao Y, Stuart DI. Crystal structure of Ebolavirus glycoprotein in complex with inhibitor 118. Worldwide Protein Data Bank. 2019. doi:10.2210/pdb6hs4/pdb

9. Shaikh F, Zhao Y, Alvarez L, Iliopoulou M, Lohans C, Schofield CJ, et al. Structure-Based in Silico Screening Identifies a Potent Ebolavirus Inhibitor from a Traditional Chinese Medicine Library. J Med Chem. 2019 Mar 28;62(6):2928–37. doi:10.1021/acs.jmedchem.8b01328

10. Gilman MSA, Rutten L, Langedijk JPM, McLellan JS. Crystal Structure of Stabilized GP from Makona Variant of Ebola Virus. Worldwide Protein Data Bank. 2020. doi:10.2210/pdb6vkm/pdb

11. Rutten L, Gilman MSA, Blokland S, Juraszek J, McLellan JS, Langedijk JPM. Structure-Based Design of Prefusion-Stabilized Filovirus Glycoprotein Trimers. Cell Rep. 2020 Mar;30(13):4540-4550.e3. doi:10.1016/j.celrep.2020.03.025

12. Abendroth J, Dranow DM, Lorimer DD, Horanyi PS, Edwards TE. Crystal Structure of Ebola zaire Envelope glycoprotein GP in complex with compound ARN0075146. Worldwide Protein Data Bank. 2021. doi:10.2210/pdb7lyd/pdb

13. Abendroth J, Fox III D, Lorimer DD, Horanyi PS, Edwards TE. Crystal Structure of Ebola zaire Envelope glycoprotein GP in complex with compound ARN0075231. Worldwide Protein Data Bank. 2023. doi:10.2210/pdb7ssq/pdb

14. Malashkevich VN, Schneider BJ, Mcnally ML, Milhollen MA, Pang JX, Kim PS. Core Structure of GP2 from Ebola Virus. Worldwide Protein Data Bank. 1999. doi:10.2210/pdb2ebo/pdb

15. Malashkevich VN, Schneider BJ, McNally ML, Milhollen MA, Pang JX, Kim PS. Core structure of the envelope glycoprotein GP2 from Ebola virus at 1.9-Å resolution. Proceedings of the National Academy of Sciences. 1999 Mar 16;96(6):2662–7. doi:10.1073/pnas.96.6.2662

16. Xiong X, Coombs P, Martin SR, Liu J, Xiao H, McCauley JW, et al. Crystal Structure of H5 (tyTy) Influenza Virus Haemagglutinin. Worldwide Protein Data Bank. 2013. doi:10.2210/pdb4bgz/pdb

17. Xiong X, Coombs PJ, Martin SR, Liu J, Xiao H, McCauley JW, et al. Receptor binding by a ferret-transmissible H5 avian influenza virus. Nature. 2013 May 24;497(7449):392–6. doi:10.1038/nature12144

18. Ni F, Kondrashkina E, Wang Q. Crystal structure of hemagglutinin of A/Taiwan/2/2013 (H6N1). Worldwide Protein Data Bank. 2015. doi:10.2210/pdb5br0/pdb

19. Ni F, Kondrashkina E, Wang Q. Structural and Functional Studies of Influenza Virus A/H6 Hemagglutinin. PLoS One. 2015 Jul 30;10(7):e0134576. doi:10.1371/journal.pone.0134576

20. Antanasijevic A, Durst MA, Lavie A, Caffrey M. Crystal Structure of a H5N1 influenza virus hemagglutinin with CBS1117. Worldwide Protein Data Bank. 2020. doi:10.2210/pdb6vmz/pdb

21. Antanasijevic A, Durst MA, Cheng H, Gaisina IN, Perez JT, Manicassamy B, et al. Structure of avian influenza hemagglutinin in complex with a small molecule entry inhibitor. Life Sci Alliance. 2020 Aug 1;3(8):e202000724. doi:10.26508/lsa.202000724

22. Bullough PA, Hughson FM, Skehel JJ, Wiley DC. STRUCTURE OF INFLUENZA HAEMAGGLUTININ AT THE PH OF MEMBRANE FUSION. Worldwide Protein Data Bank. 1995. doi:10.2210/pdb1htm/pdb

23. Bullough PA, Hughson FM, Skehel JJ, Wiley DC. Structure of influenza haemagglutinin at the pH of membrane fusion. Nature. 1994 Sep 1;371(6492):37–43. doi:10.1038/371037a0

24. Ni F, Chen X, Shen J, Wang Q. Structure of influenza B virus hemagglutinin at membrane fusion pH. Worldwide Protein Data Bank. 2014. doi:10.2210/pdb4nkj/pdb

25. Ni F, Chen X, Shen J, Wang Q. Structural Insights into the Membrane Fusion Mechanism Mediated by Influenza Virus Hemagglutinin. Biochemistry. 2014 Feb 11;53(5):846–54. doi:10.1021/bi401525h

26. Benton DJ, Rosenthal PB. Signal Subtracted Extended Intermediate form of X-31 Influenza Haemagglutinin at pH 5 (State IV). Worldwide Protein Data Bank. 2020. doi:10.2210/pdb6y5l/pdb

27. Benton DJ, Gamblin SJ, Rosenthal PB, Skehel JJ. Structural transitions in influenza haemagglutinin at membrane fusion pH. Nature. 2020 Jul 2;583(7814):150–3. doi:10.1038/s41586-020-2333-6

28. Hastie KM, Enriquez AS. Structure of Lassa virus glycoprotein bound to Fab 18.5C and Fab 36.1F. Worldwide Protein Data Bank. 2022. doi:10.2210/pdb7s8h/pdb

29. Enriquez AS, Buck TK, Li H, Norris MJ, Moon-Walker A, Zandonatti MA, et al. Delineating the mechanism of anti-Lassa virus GPC-A neutralizing antibodies. Cell Rep. 2022 May;39(8):110841. doi:10.1016/j.celrep.2022.110841

30. Antanasijevic A, Brouwer PJM, Ward AB. Lassa virus glycoprotein construct(Josiah GPCysR4) recovered from GPC-I53-50 nanoparticle by localized reconstruction. Worldwide Protein Data Bank. 2022. doi:10.2210/pdb7sgd/pdb

31. Brouwer PJM, Antanasijevic A, Ronk AJ, Müller-Kräuter H, Watanabe Y, Claireaux M, et al. Lassa virus glycoprotein nanoparticles elicit neutralizing antibody responses and protection. Cell Host Microbe. 2022 Dec;30(12):1759-1772.e12. doi:10.1016/j.chom.2022.10.018

32. Buck TK, Enriquez AS, Hastie KM. Lineage I (Pinneo) Lassa virus glycoprotein bound to 18.5C-M30 Fab. Worldwide Protein Data Bank. 2022. doi:10.2210/pdb7ul7/pdb

33. Buck TK, Enriquez AS, Schendel SL, Zandonatti MA, Harkins SS, Li H, et al. Neutralizing Antibodies against Lassa Virus Lineage I. mBio. 2022 Aug 30;13(4). doi:10.1128/mbio.01278-22

34. Hastie KM, Zandonatti MA, Kleinfelter LM, Rowland ML, Rowland MM, Chandra K, et al. Structural basis for antibody-mediated neutralization of Lassa virus. Worldwide Protein Data Bank. 2017. doi:10.2210/pdb5vk2/pdb

35. Hastie KM, Zandonatti MA, Kleinfelter LM, Heinrich ML, Rowland MM, Chandran K, et al. Structural basis for antibody-mediated neutralization of Lassa virus. Science (1979). 2017 Jun 2;356(6341):923–8. doi:10.1126/science.aam7260

36. Perrett HR, Ward AB. Structure of lineage IV Lassa virus glycoprotein complex (strain Josiah). Worldwide Protein Data Bank. 2023. doi:10.2210/pdb8ejd/pdb

37. Perrett HR, Brouwer PJM, Hurtado J, Newby ML, Liu L, Müller-Kräuter H, et al. Structural conservation of Lassa virus glycoproteins and recognition by neutralizing antibodies. Cell Rep. 2023 May;42(5):112524. doi:10.1016/j.celrep.2023.112524

38. Perrett HR, Ward AB. Structure of lineage V Lassa virus glycoprotein complex (strain Soromba-R). Worldwide Protein Data Bank. 2023. doi:10.2210/pdb8ejf/pdb

39. Shulman A, Diskin R. Crystal structure of GP2 from Lassa virus in a post fusion conformation. Worldwide Protein Data Bank. 2018. doi:10.2210/pdb5omi/pdb

40. Shulman A, Katz M, Cohen-Dvashi H, Greenblatt HM, Levy Y, Diskin R. Variations in Core Packing of GP2 from Old World Mammarenaviruses in their Post-Fusion Conformations Affect Membrane-Fusion Efficiencies. J Mol Biol. 2019 May;431(11):2095–111. doi:10.1016/j.jmb.2019.04.012

41. Zhu Y, Zhang X, Chen B, Ye S, Zhang R. Crystal structure of LASV-GP2 in a post fusion conformation. Worldwide Protein Data Bank. 2019. doi:10.2210/pdb6jgy/pdb

42. Zhang X, Wang C, Chen B, Wang Q, Xu W, Ye S, et al. Crystal Structure of Refolding Fusion Core of Lassa Virus GP2 and Design of Lassa Virus Fusion Inhibitors. Front Microbiol. 2019 Aug 13;10. doi:10.3389/fmicb.2019.01829

43. Hashiguchi T, Fusco ML, Hastie KM, Bomholdt ZA, Lee JE, Flyak AI, et al. Crystal structure of Marburg virus GP in complex with the human survivor antibody MR78. Worldwide Protein Data Bank. 2017. doi:10.2210/pdb5uqy/pdb

44. Hashiguchi T, Fusco ML, Bornholdt ZA, Lee JE, Flyak AI, Matsuoka R, et al. Structural Basis for Marburg Virus Neutralization by a Cross-Reactive Human Antibody. Cell. 2015 Feb;160(5):904–12. doi:10.1016/j.cell.2015.01.041

45. King LB, Fusco ML, Flyak AI, Ilinykh PA, Huang K, Gunn B, et al. Therapeutic human monoclonal antibody MR191 bound to a marburgvirus glycoprotein. Worldwide Protein Data Bank. 2018. doi:10.2210/pdb6bp2/pdb

46. King LB, Fusco ML, Flyak AI, Ilinykh PA, Huang K, Gunn B, et al. The Marburgvirus-Neutralizing Human Monoclonal Antibody MR191 Targets a Conserved Site to Block Virus Receptor Binding. Cell Host Microbe. 2018 Jan;23(1):101-109.e4. doi:10.1016/j.chom.2017.12.003

47. Malashkevich VN, Koellhoffer JF, Harrison JS, Toro R, Bhosle RC, Chandran K, et al. Crystal structure of the Marburg Virus GP2 ectodomain in its post-fusion conformation. Worldwide Protein Data Bank. 2012. doi:10.2210/pdb4g2k/pdb

48. Koellhoffer JF, Malashkevich VN, Harrison JS, Toro R, Bhosle RC, Chandran K, et al. Crystal Structure of the Marburg Virus GP2 Core Domain in Its Postfusion Conformation. Biochemistry. 2012 Oct 2;51(39):7665–75. doi:10.1021/bi300976m

49. Battles MB, McLellan JS, Arnoult E, Roymans D, Langedijk JP. Crystal Structure of Prefusion RSV F Glycoprotein Fusion Inhibitor Resistance Mutant D489Y. Worldwide Protein Data Bank. 2015. doi:10.2210/pdb5ea8/pdb

50. Battles MB, Langedijk JP, Furmanova-Hollenstein P, Chaiwatpongsakorn S, Costello HM, Kwanten L, et al. Molecular mechanism of respiratory syncytial virus fusion inhibitors. Nat Chem Biol. 2016 Feb 7;12(2):87–93. doi:10.1038/nchembio.1982

51. McLellan JS. Crystal Structure of RSV F B9320 DS-Cav1. Worldwide Protein Data Bank. 2017. doi:10.2210/pdb5ude/pdb

52. Zhu Q, McLellan JS, Kallewaard NL, Ulbrandt ND, Palaszynski S, Zhang J, et al. A highly potent extended half-life antibody as a potential RSV vaccine surrogate for all infants. Sci Transl Med. 2017 May 3;9(388). doi:10.1126/scitranslmed.aaj1928

53. Battles MB, McLellan JS. Crystal structure of human metapneumovirus fusion glycoprotein stabilized in the prefusion state. Worldwide Protein Data Bank. 2017. doi:10.2210/pdb5wb0/pdb

54. Battles MB, Más V, Olmedillas E, Cano O, Vázquez M, Rodríguez L, et al. Structure and immunogenicity of pre-fusion-stabilized human metapneumovirus F glycoprotein. Nat Commun. 2017 Nov 16;8(1):1528. doi:10.1038/s41467-017-01708-9

55. Battles MB, McLellan JS. Crystal Structure Of Human Respiratory Syncytial Virus Fusion Glycoprotein Inhibitor Escape Variant F140i Stabilized In The Prefusion State. Worldwide Protein Data Bank. 2019. doi:10.2210/pdb6ead/pdb

56. Mclellan JS, Joyce MG, Stewart-Jones GBE, Sastry M, Yang Y, Graham BS, et al. Crystal Structure of Prefusion-stabilized RSV F Variant Cav1 at pH 5.5. Worldwide Protein Data Bank. 2013. doi:10.2210/pdb4mms/pdb

57. McLellan JS, Chen M, Joyce MG, Sastry M, Stewart-Jones GBE, Yang Y, et al. Structure-Based Design of a Fusion Glycoprotein Vaccine for Respiratory Syncytial Virus. Science (1979). 2013 Nov;342(6158):592–8. doi:10.1126/science.1243283

58. Swanson KA, Settembre EC, Shaw CA, Dey AK, Rappuoli R, Mandl CW, et al. Structural basis for immunization with post-fusion RSV F to elicit high neutralizing antibody titers. Worldwide Protein Data Bank. 2011. doi:10.2210/pdb3rki/pdb

59. Swanson KA, Settembre EC, Shaw CA, Dey AK, Rappuoli R, Mandl CW, et al. Structural basis for immunization with postfusion respiratory syncytial virus fusion F glycoprotein (RSV F) to elicit high neutralizing antibody titers. Proceedings of the National Academy of Sciences. 2011 Jun 7;108(23):9619–24. doi:10.1073/pnas.1106536108

60. McLellan JS, Yongping Y, Graham BS, Kwong PD. Structure of the RSV F protein in the post-fusion conformation. Worldwide Protein Data Bank. 2011. doi:10.2210/pdb3rrr/pdb

61. McLellan JS, Yang Y, Graham BS, Kwong PD. Structure of Respiratory Syncytial Virus Fusion Glycoprotein in the Postfusion Conformation Reveals Preservation of Neutralizing Epitopes. J Virol. 2011 Aug;85(15):7788–96. doi:10.1128/JVI.00555-11
