# Supplementary material for: Prediction of pre- and postfusion conformations of class I fusion proteins with AlphaFold2
Source: PLoS One. 2026 Jun 16;21(6):e0351662. doi: 10.1371/journal.pone.0351662 (PMC13271458; doi:10.1371/journal.pone.0351662)
Supplement: S4 Table — (PDF) [file pone.0351662.s004.pdf]

S4 Table. Input sequences of GP2 for the canonical benchmark set designs.

>EBOV_3CSY_Zaire-1976
EAIVNAQPKCNPNLHYWTTQDEGAAIGLAWIPYFGPAAEGIYTEGLMHNQDGLICGLRQLANETTQALQ
LFLRATTELRTFSILNRKAIDFLLQRWGGTCHILGPDCCIEPHDWTKNITDKIDQIIHDFVD:
EAIVNAQPKCNPNLHYWTTQDEGAAIGLAWIPYFGPAAEGIYTEGLMHNQDGLICGLRQLANETTQALQ
LFLRATTELRTFSILNRKAIDFLLQRWGGTCHILGPDCCIEPHDWTKNITDKIDQIIHDFVD:
EAIVNAQPKCNPNLHYWTTQDEGAAIGLAWIPYFGPAAEGIYTEGLMHNQDGLICGLRQLANETTQALQ
LFLRATTELRTFSILNRKAIDFLLQRWGGTCHILGPDCCIEPHDWTKNITDKIDQIIHDFVD

>HA_A_Aichi_2_1968_H3N2
GLFGAIAGFIENGWEGMIDGWYGFRHQNSEGTGQAADLKSTQAAIDQINGKLNRVIEKTNEKFHQIEKE
FSEVEGRIQDLEKYVEDTKIDLWSYNAELLVALENQHTIDLTDSEMNKLFEKTRRQLRENAEEMGNGCF
KIYHKCDNACIESIRNGTYDHDVYRDEALNNRFQIKG:
GLFGAIAGFIENGWEGMIDGWYGFRHQNSEGTGQAADLKSTQAAIDQINGKLNRVIEKTNEKFHQIEKE
FSEVEGRIQDLEKYVEDTKIDLWSYNAELLVALENQHTIDLTDSEMNKLFEKTRRQLRENAEEMGNGCF
KIYHKCDNACIESIRNGTYDHDVYRDEALNNRFQIKG:
GLFGAIAGFIENGWEGMIDGWYGFRHQNSEGTGQAADLKSTQAAIDQINGKLNRVIEKTNEKFHQIEKE
FSEVEGRIQDLEKYVEDTKIDLWSYNAELLVALENQHTIDLTDSEMNKLFEKTRRQLRENAEEMGNGCF
KIYHKCDNACIESIRNGTYDHDVYRDEALNNRFQIK

>LASV1_Josiah_1976
GTFTWTLSDSEGKDTPGGYCLTRWMLIEAELKCFGNTAVAKCNEKHDEEFCDMLRLFDFNKQAIQRLK
AEAQMSIQLINKAVNALINDQLIMKNHLRDIMGIPYCNYSKYWYLNHTTTGRTSLPKCWLVSNGSYLNE
THFSDDIEQQADNMITEMLQKEYMERQGKTPLGLVDLFVFSTSFYLISIFLHLVKIPTHRHIVGKSCPKPH
RLNHMGICSCGLYKQPGVPVKWKR:
GTFTWTLSDSEGKDTPGGYCLTRWMLIEAELKCFGNTAVAKCNEKHDEEFCDMLRLFDFNKQAIQRLK
AEAQMSIQLINKAVNALINDQLIMKNHLRDIMGIPYCNYSKYWYLNHTTTGRTSLPKCWLVSNGSYLNE
THFSDDIEQQADNMITEMLQKEYMERQGKTPLGLVDLFVFSTSFYLISIFLHLVKIPTHRHIVGKSCPKPH
RLNHMGICSCGLYKQPGVPVKWKR:
GTFTWTLSDSEGKDTPGGYCLTRWMLIEAELKCFGNTAVAKCNEKHDEEFCDMLRLFDFNKQAIQRLK
AEAQMSIQLINKAVNALINDQLIMKNHLRDIMGIPYCNYSKYWYLNHTTTGRTSLPKCWLVSNGSYLNE
THFSDDIEQQADNMITEMLQKEYMERQGKTPLGLVDLFVFSTSFYLISIFLHLVKIPTHRHIVGKSCPKPH
RLNHMGICSCGLYKQPGVPVKWKR

>LASV2_Josiah_1976
GTFTWTLSDSEGKDTPGGYCLTRWMLIEAELKCFGNTAVAKCNEKHDEEFCDMLRLFDFNKQAIQRLK
APAQTSIQLINKAVNALINDQLIMKNHLRDIMCIPYCNYSKYWYLNHTTTGRTSLPKCWLVSNGSYLNE
THFSDDIEQQADNMITEMLQKEYMERQGKTPLGLVD:
GTFTWTLSDSEGKDTPGGYCLTRWMLIEAELKCFGNTAVAKCNEKHDEEFCDMLRLFDFNKQAIQRLK
APAQTSIQLINKAVNALINDQLIMKNHLRDIMCIPYCNYSKYWYLNHTTTGRTSLPKCWLVSNGSYLNE
THFSDDIEQQADNMITEMLQKEYMERQGKTPLGLVD:
GTFTWTLSDSEGKDTPGGYCLTRWMLIEAELKCFGNTAVAKCNEKHDEEFCDMLRLFDFNKQAIQRLK
APAQTSIQLINKAVNALINDQLIMKNHLRDIMCIPYCNYSKYWYLNHTTTGRTSLPKCWLVSNGSYLNE
THFSDDIEQQADNMITEMLQKEYMERQGKTPLGLVD

>MARV_6BP2_RAVN-87
SILAKEGDIGPNLDGLINTEIDFDPIPNTETIFDESPSFNTSTNEEQHTPPNISLTFSYFPDKNGDTAYSGENE
NDCDAELRIWSVQEDDLAAGLSWIPFFGPGIEGLYTAGLIKNQNNLVCRLRRLANQTAKSLELLLRVTTE
ERTFSLINRHAIDFLLTRWGGTCKVLGPDCCIGIEDLSKNISEQIDKIRKDEQKEETG:
SILAKEGDIGPNLDGLINTEIDFDPIPNTETIFDESPSFNTSTNEEQHTPPNISLTFSYFPDKNGDTAYSGENE
NDCDAELRIWSVQEDDLAAGLSWIPFFGPGIEGLYTAGLIKNQNNLVCRLRRLANQTAKSLELLLRVTTE
ERTFSLINRHAIDFLLTRWGGTCKVLGPDCCIGIEDLSKNISEQIDKIRKDEQKEETG:
SILAKEGDIGPNLDGLINTEIDFDPIPNTETIFDESPSFNTSTNEEQHTPPNISLTFSYFPDKNGDTAYSGENE
NDCDAELRIWSVQEDDLAAGLSWIPFFGPGIEGLYTAGLIKNQNNLVCRLRRLANQTAKSLELLLRVTTE
ERTFSLINRHAIDFLLTRWGGTCKVLGPDCCIGIEDLSKNISEQIDKIRKDEQKEETG
