# Supplementary material for: Prediction of pre- and postfusion conformations of class I fusion proteins with AlphaFold2
Source: PLoS One. 2026 Jun 16;21(6):e0351662. doi: 10.1371/journal.pone.0351662 (PMC13271458; doi:10.1371/journal.pone.0351662)
Supplement: S5 Table — (PDF) [file pone.0351662.s005.pdf]

S5 Table. Input sequences of GP2 for the real-world benchmark set designs.

>JUNV_NP899218 | NCBI Protein: NP_899218.1 | UniProtKB: Q6UY73 | Junín virus<br>AFFSWSLTDSSGKDTPGGYCLEEWMLVAAKMKCFGNTAVAKCNLNHDSEFCDMLRLFDYNKNAIKTL<br>NDETKKQVNLMGQTINALISDNLLMKNKIRELMSVPYCNYTKFWYVNHTLSGQHSLPRCWLIKNNSYL<br>NISDFRNDWILESDFLISEMLSKEYSDRQGKTPLTLVDICFWSTVFFTASLFLHLVGIPTHRHIRGEACPLP<br>HRLNSLGGCRCGKYPNLKKPTVWRRGH:<br>AFFSWSLTDSSGKDTPGGYCLEEWMLVAAKMKCFGNTAVAKCNLNHDSEFCDMLRLFDYNKNAIKTL<br>NDETKKQVNLMGQTINALISDNLLMKNKIRELMSVPYCNYTKFWYVNHTLSGQHSLPRCWLIKNNSYL<br>NISDFRNDWILESDFLISEMLSKEYSDRQGKTPLTLVDICFWSTVFFTASLFLHLVGIPTHRHIRGEACPLP<br>HRLNSLGGCRCGKYPNLKKPTVWRRGH:<br>AFFSWSLTDSSGKDTPGGYCLEEWMLVAAKMKCFGNTAVAKCNLNHDSEFCDMLRLFDYNKNAIKTL<br>NDETKKQVNLMGQTINALISDNLLMKNKIRELMSVPYCNYTKFWYVNHTLSGQHSLPRCWLIKNNSYL<br>NISDFRNDWILESDFLISEMLSKEYSDRQGKTPLTLVDICFWSTVFFTASLFLHLVGIPTHRHIRGEACPLP<br>HRLNSLGGCRCGKYPNLKKPTVWRRGH
>MACV_NP899212 | NCBI Protein: NP_899212.1 | UniProtKB: Q8AZ57 | Machupo virus<br>AFFSWSLTDSSGKDMPGGYCLEEWMLIAAKMKCFGNTAVAKCNQNHDSEFCDMLRLFDYNKNAIKTL<br>NDESKKEINLLSQTVNALISDNLLMKNKIKELMSIPYCNYTKFWYVNHTLTGQHTLPRCWLIRNGSYLN<br>TSEFRNDWILESDHLISEMLSKEYAERQGKTPITLVDICFWSTIFFTASLFLHLVGIPTHRHLKGEACPLPH<br>KLDSFGGCRCGKYPRLKKPTIWHKRH:<br>AFFSWSLTDSSGKDMPGGYCLEEWMLIAAKMKCFGNTAVAKCNQNHDSEFCDMLRLFDYNKNAIKTL<br>NDESKKEINLLSQTVNALISDNLLMKNKIKELMSIPYCNYTKFWYVNHTLTGQHTLPRCWLIRNGSYLN<br>TSEFRNDWILESDHLISEMLSKEYAERQGKTPITLVDICFWSTIFFTASLFLHLVGIPTHRHLKGEACPLPH<br>KLDSFGGCRCGKYPRLKKPTIWHKRH:<br>AFFSWSLTDSSGKDMPGGYCLEEWMLIAAKMKCFGNTAVAKCNQNHDSEFCDMLRLFDYNKNAIKTL<br>NDESKKEINLLSQTVNALISDNLLMKNKIKELMSIPYCNYTKFWYVNHTLTGQHTLPRCWLIRNGSYLN<br>TSEFRNDWILESDHLISEMLSKEYAERQGKTPITLVDICFWSTIFFTASLFLHLVGIPTHRHLKGEACPLPH<br>KLDSFGGCRCGKYPRLKKPTIWHKRH
>CHAVB-2003 | NCBI Protein: YP_001816782.1 | UniProtKB: B2C4J0 | Chapare virus<br>GVFTWTITDAAGNDMPGGYCLERWMLVTSDLKCFGNTALAKCNLNHDSEFCDMLKLFEFNKKAIESLN<br>DNTKNKVNLLTHSINALISDNLLMKNRLKELLDTPYCNYTKFWYVNHTITGEHSLPRCWMVKNNSYLN<br>ESEFRNDWILESDHLLSEMLNKEYFDRQGKTPITLVD:<br>GVFTWTITDAAGNDMPGGYCLERWMLVTSDLKCFGNTALAKCNLNHDSEFCDMLKLFEFNKKAIESLN<br>DNTKNKVNLLTHSINALISDNLLMKNRLKELLDTPYCNYTKFWYVNHTITGEHSLPRCWMVKNNSYLN<br>ESEFRNDWILESDHLLSEMLNKEYFDRQGKTPITLVD:<br>GVFTWTITDAAGNDMPGGYCLERWMLVTSDLKCFGNTALAKCNLNHDSEFCDMLKLFEFNKKAIESLN<br>DNTKNKVNLLTHSINALISDNLLMKNRLKELLDTPYCNYTKFWYVNHTITGEHSLPRCWMVKNNSYLN<br>ESEFRNDWILESDHLLSEMLNKEYFDRQGKTPITLVD
>LUJV | NCBI Protein: ACR56359.1 | UniProtKB: C5ILC1 | Lujo virus<br>KLFQWSLSDETGSPLPGGHCLERWLIFASDIKCFDNAAIAKCNKEHDEEFCDMLRLFDYNKASIAKLRGE<br>ASSSINLLSGRINAIISDTLLMRSSLKRLMGIPYCNYTKFWYLNHTKLGIHSLPRCWLVSNGSYLNETKFT<br>HDMEDEADKLLTEMLKKEYVRRQEKTPITLMDILMFSVSFYMFSVTLCICNIPTHRHITGLPCPKPHRLR<br>KNGTCACGFFKSINRSTGWAKH:<br>KLFQWSLSDETGSPLPGGHCLERWLIFASDIKCFDNAAIAKCNKEHDEEFCDMLRLFDYNKASIAKLRGE<br>ASSSINLLSGRINAIISDTLLMRSSLKRLMGIPYCNYTKFWYLNHTKLGIHSLPRCWLVSNGSYLNETKFT<br>HDMEDEADKLLTEMLKKEYVRRQEKTPITLMDILMFSVSFYMFSVTLCICNIPTHRHITGLPCPKPHRLR<br>KNGTCACGFFKSINRSTGWAKH:<br>KLFQWSLSDETGSPLPGGHCLERWLIFASDIKCFDNAAIAKCNKEHDEEFCDMLRLFDYNKASIAKLRGE<br>ASSSINLLSGRINAIISDTLLMRSSLKRLMGIPYCNYTKFWYLNHTKLGIHSLPRCWLVSNGSYLNETKFT<br>HDMEDEADKLLTEMLKKEYVRRQEKTPITLMDILMFSVSFYMFSVTLCICNIPTHRHITGLPCPKPHRLR<br>KNGTCACGFFKSINRSTGWAKH
