# Supplementary material for: Prediction of pre- and postfusion conformations of class I fusion proteins with AlphaFold2
Source: PLoS One. 2026 Jun 16;21(6):e0351662. doi: 10.1371/journal.pone.0351662 (PMC13271458; doi:10.1371/journal.pone.0351662)
Supplement: S6 Table — Structures were obtained from the Protein Data Bank (PDB). (PDF) [file pone.0351662.s006.pdf]

S6 Table. Reference structures of the real-world benchmark set for the prefusion specific analyses. Structures were obtained from the Protein Data Bank (PDB) (1).

VIRUS	PDB	CONFORMATION	PDB DOI	PAPER DOI
JUNV	9GHJ	PRE	(2)	(3)
LUJV	8P4T	PRE	(4)	(5)
MACV	9GHI	PRE	(6)	(7)

*PRE=Prefusion conformation

References

1. Berman HM. The Protein Data Bank. Nucleic Acids Res. 2000 Jan 1;28(1):235–42. doi:10.1093/nar/28.1.235
2. Bowden TA, Paesen GC. Junin virus GP1-GP2 heterodimer in complex with Fab of JUN1. Worldwide Protein Data Bank. 2025. doi:10.2210/pdb9ghj/pdb
3. Paesen GC, Ng WM, Kimuda S, Sutton G, Doores KJ, Bowden TA. Structure and stabilization of the antigenic glycoprotein building blocks of the New World mammarenavirus spike complex. mBio. 2025 Jul 9;16(7). doi:10.1128/mbio.01076-25
4. Eilon-Ashkenazy M, Diskin R. The spike complex of the Lujo Virus. Worldwide Protein Data Bank. 2024. doi:10.2210/pdb8p4t/pdb
5. Eilon-Ashkenazy M, Cohen-Dvashi H, Borni S, Shaked R, Calinsky R, Levy Y, et al. The structure of the Lujo virus spike complex. Nat Commun. 2024 Aug 21;15(1):7175. doi:10.1038/s41467-024-51606-0
6. Bowden TA, Paesen GC. Machupo virus GP1-GP2 heterodimer in complex with Fab of MAC1. Worldwide Protein Data Bank. 2025. doi:10.2210/pdb9ghi/pdb
7. Paesen GC, Ng WM, Kimuda S, Sutton G, Doores KJ, Bowden TA. Structure and stabilization of the antigenic glycoprotein building blocks of the New World mammarenavirus spike complex. mBio. 2025 Jul 9;16(7). doi:10.1128/mbio.01076-25
