# Supplementary figures and images for: Prediction of pre- and postfusion conformations of class I fusion proteins with AlphaFold2
Source: PLoS One. 2026 Jun 16;21(6):e0351662. doi: 10.1371/journal.pone.0351662 (PMC13271458; doi:10.1371/journal.pone.0351662)

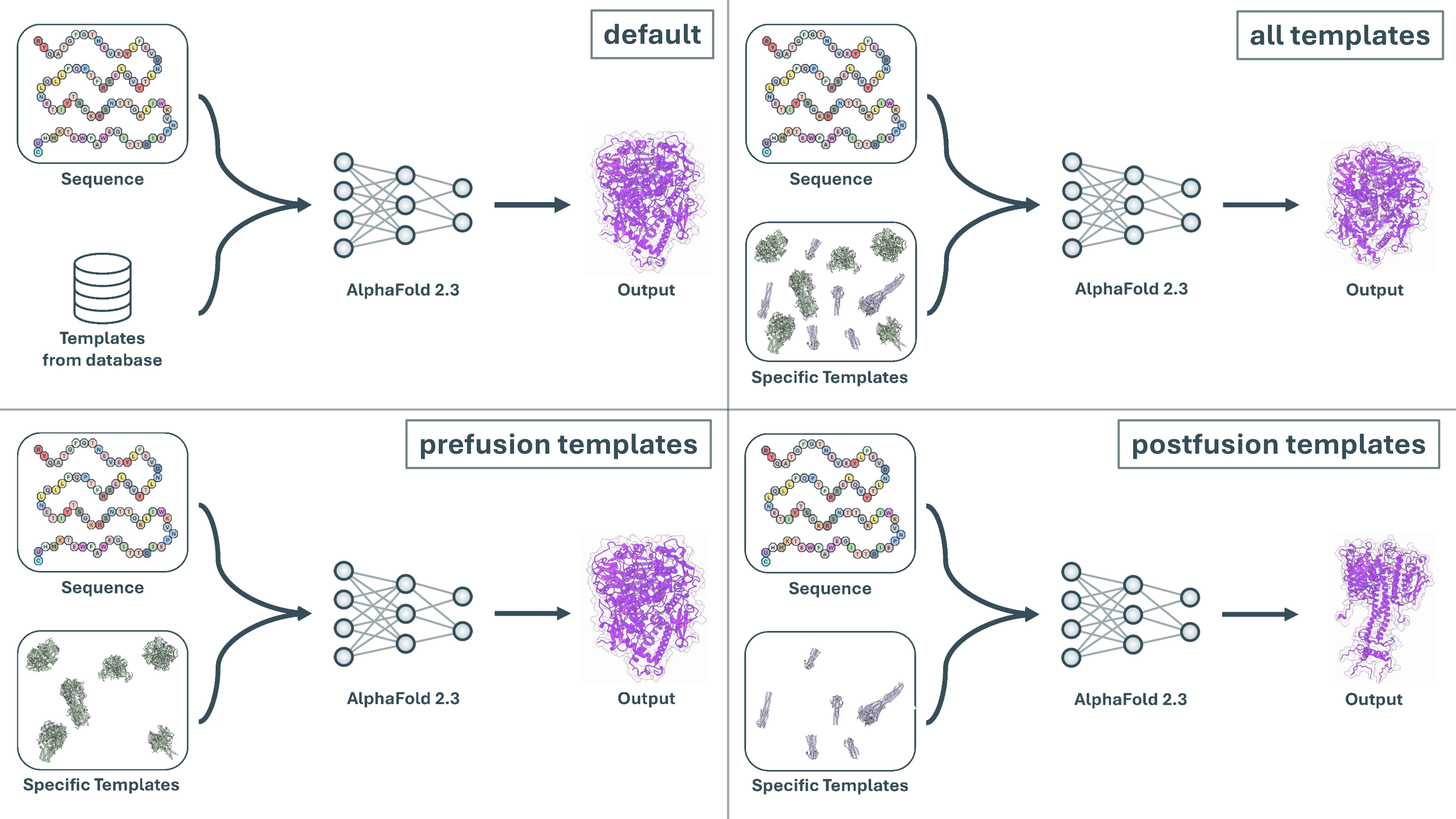

Supplement: S1 Fig — (TIF) [file pone.0351662.s007.tif]

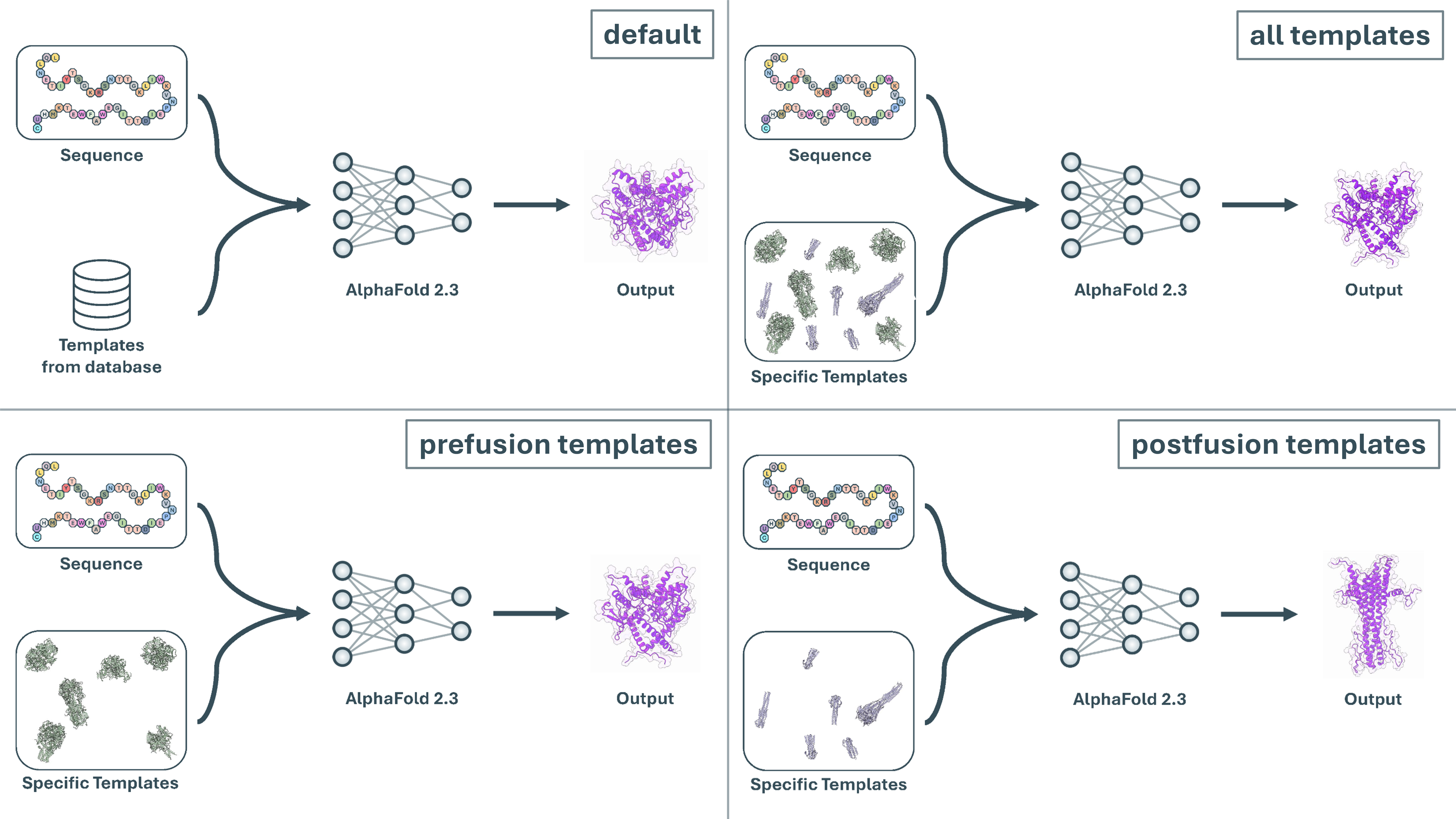

Supplement: S2 Fig — (TIF) [file pone.0351662.s008.tif]

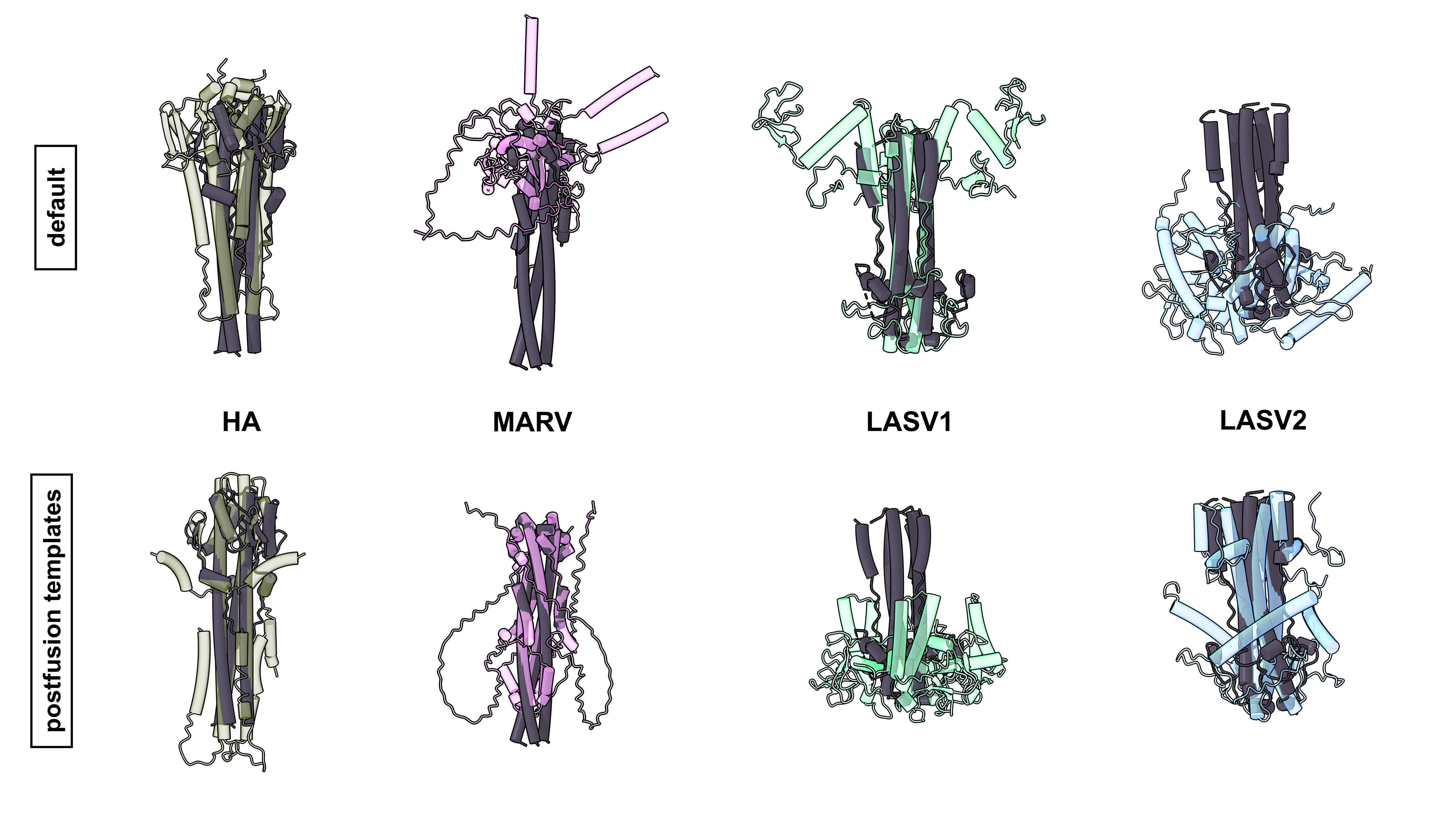

Supplement: S3 Fig — TM-score analyses for the postfusion states were performed using the corresponding experimentally determined postfusion structures as references. The models with the highest TM-scores are shown aligned with their respective postfusion reference structures, displayed in grey (respective PDB IDs as following: HA: 1HTM, MARV: 4G2K, LASV1: 5OMI, LASV2: 6JGY), while the predicted models are shown in transparent colors. (TIF) [file pone.0351662.s009.tif]

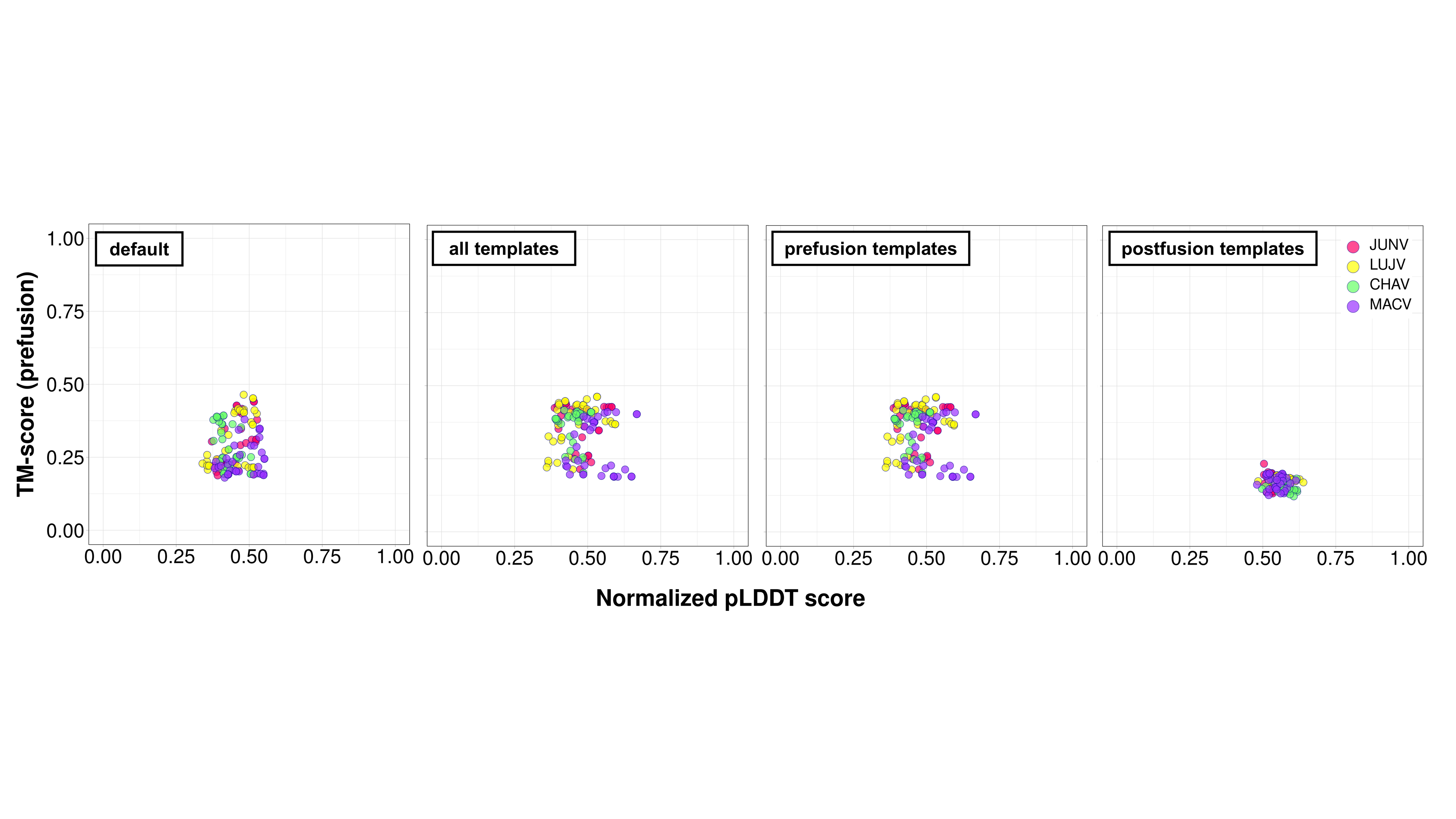

Supplement: S4 Fig — TM-score analyses for the prefusion states were conducted using their respective experimentally determined prefusion structures, except for the CHAV analysis, which utilized the JUNV structure due to the absence of an experimentally determined prefusion state for the CHAV. TM-score >=0.45 signifies similar overall structural topology. pLDDT score is considered as; very low, 0–50; low, 50–70; high, 70–90; and very high, 90–100. (TIF) [file pone.0351662.s010.tif]

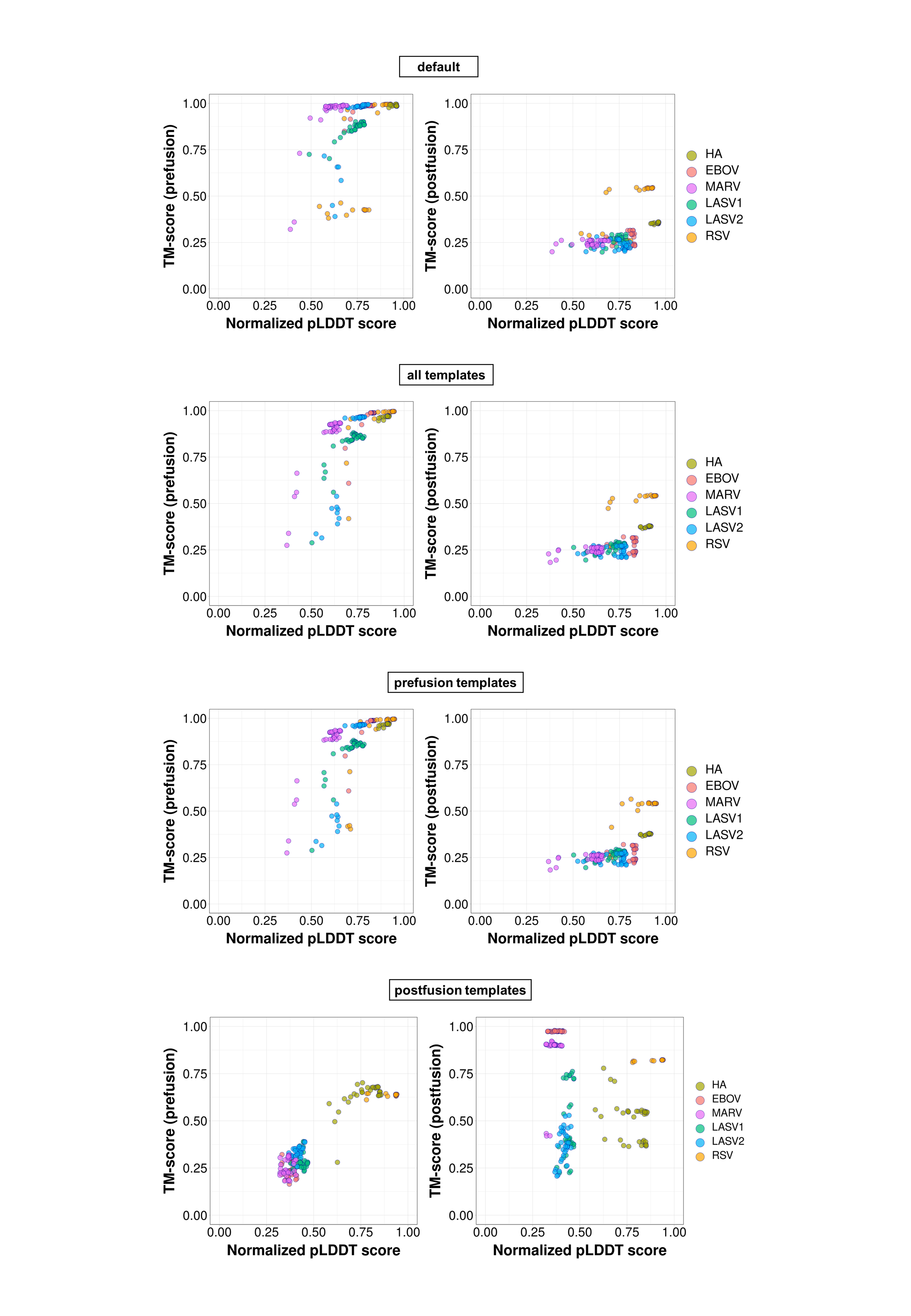

Supplement: S5 Fig — TM-score >=0.45 signifies similar overall structural topology. pLDDT score is considered as; very low, 0–0.5; low, 0.5–0.7; high, 0.7–0.9; and very high, 0.9–1. (TIF) [file pone.0351662.s011.tif]

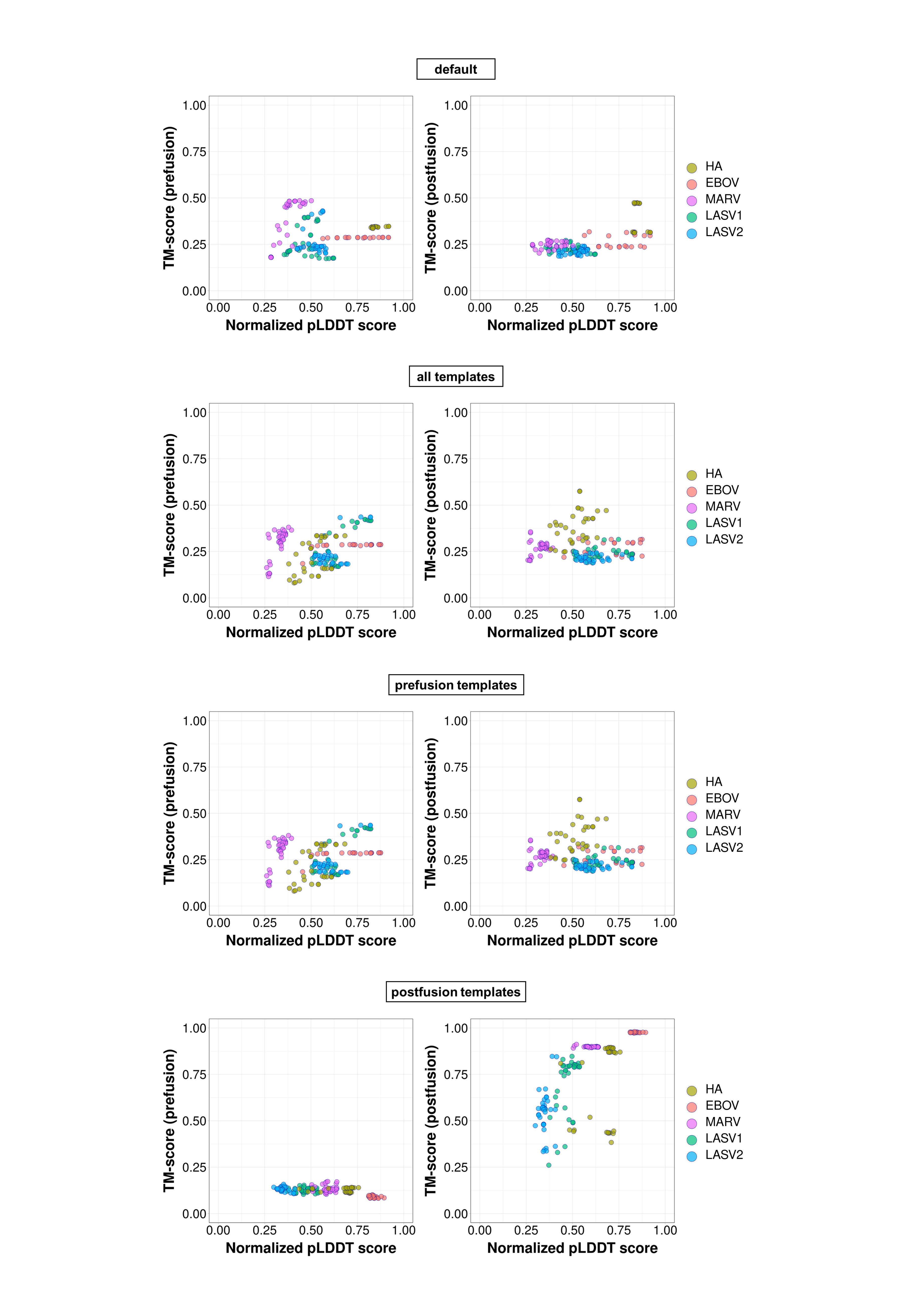

Supplement: S6 Fig — TM-score >=0.45 signifies similar overall structural topology. pLDDT score is considered as; very low, 0–0.5; low, 0.5–0.7; high, 0.7–0.9; and very high, 0.9–1. (TIF) [file pone.0351662.s012.tif]

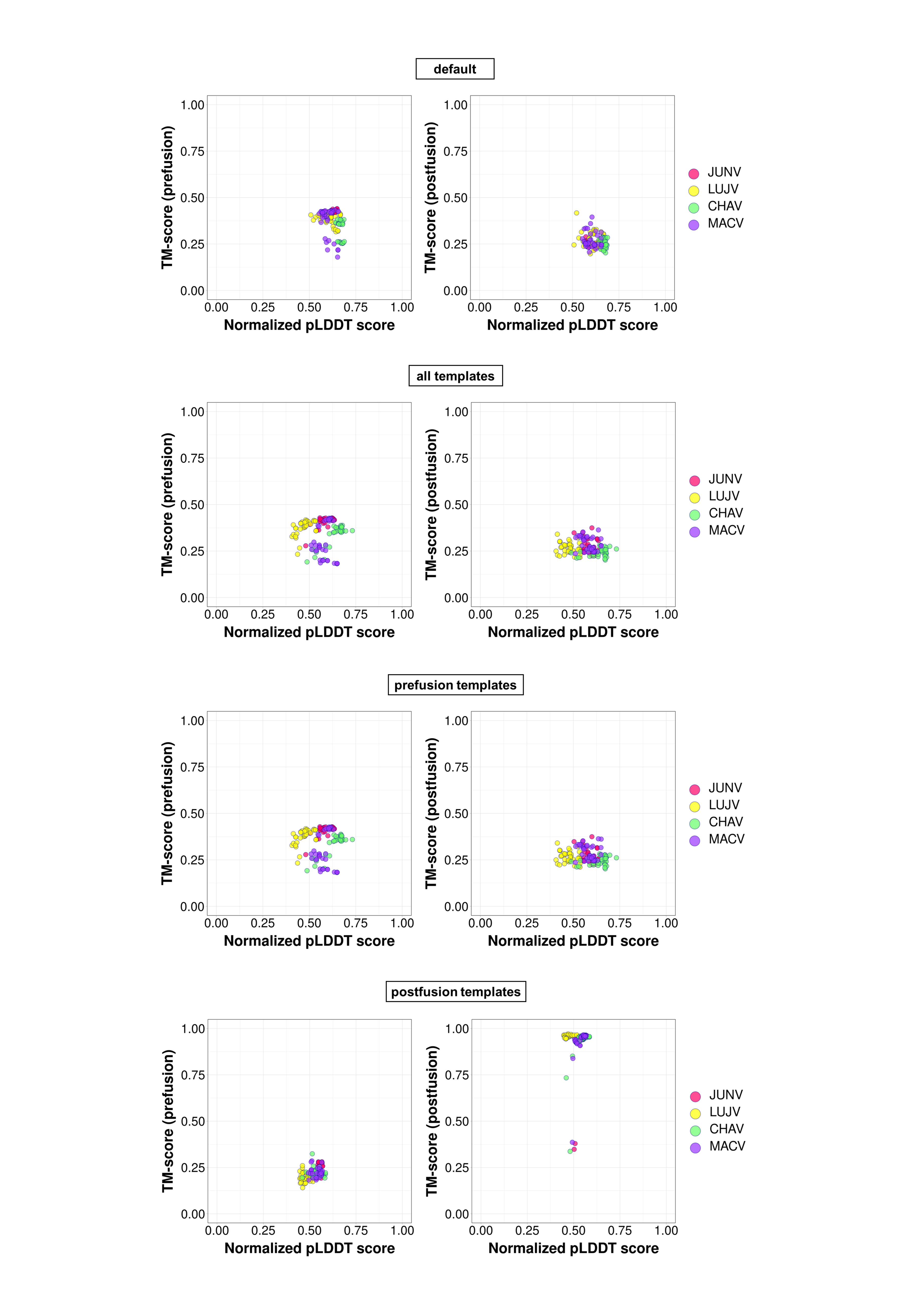

Supplement: S7 Fig — TM-score analyses for pre- and postfusion states were conducted using the LASV GPC pre- and postfusion structure, as experimentally determined postfusion structures are not available for the GP of all viruses. TM-score >=0.45 signifies similar overall structural topology. pLDDT score is considered as; very low, 0–0.5; low, 0.5–0.7; high, 0.7–0.9; and very high, 0.9–1. (TIF) [file pone.0351662.s013.tif]

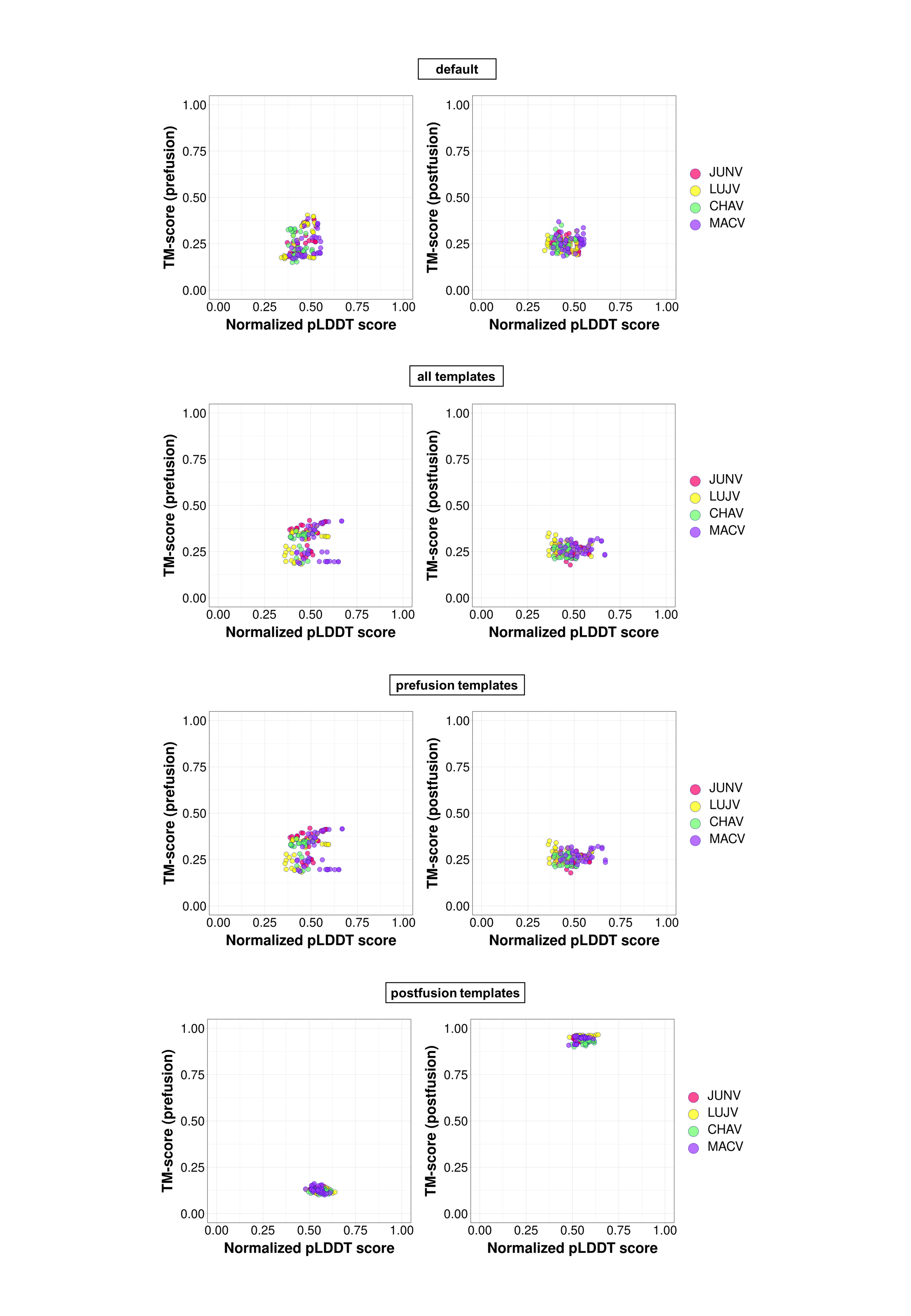

Supplement: S8 Fig — TM-score analyses for pre- and postfusion states were conducted using the LASV GPC pre- and postfusion structure, as experimentally determined postfusion structures are not available for the GP of all viruses. TM-score >=0.45 signifies similar overall structural topology. pLDDT score is considered as; very low, 0–0.5; low, 0.5–0.7; high, 0.7–0.9; and very high, 0.9–1. (TIF) [file pone.0351662.s014.tif]
